# Surface passivation extends single and biexciton lifetimes of InP quantum dots†
†Electronic supplementary information (ESI) available: Experimental and calculation details, additional sample characterization, and spectroscopic data. See DOI: 10.1039/d0sc01039a


**DOI:** 10.1039/d0sc01039a

**Published:** 2020-05-18

**Authors:** Wenxing Yang, Yawei Yang, Alexey L. Kaledin, Sheng He, Tao Jin, James R. McBride, Tianquan Lian

**Affiliations:** a Department of Chemistry , Emory University , 1515 Dickey Drive Northeast , Atlanta , Georgia 30322 , USA . Email: wenxing.yang@emory.edu ; Email: tlian@emory.edu; b Department of Chemistry − Ångström Laboratory , Physical Chemistry , Uppsala University , SE-75120 Uppsala , Sweden; c Electronic Materials Research Laboratory , Key Laboratory of the Ministry of Education , International Center for Dielectric Research , Shaanxi Engineering Research Center of Advanced Energy Materials and Devices , School of Electronic Science and Engineering , Xi'an Jiaotong University , Xi'an 710049 , Shaanxi , P. R. China; d Cherry L. Emerson Center for Scientific Computation , Emory University , 1515 Dickey Drive , Atlanta , GA 30322 , USA; e Department of Chemistry , The Vanderbilt Institute of Nanoscale Science and Engineering , Vanderbilt University , Nashville , TN 37235 , USA

## Abstract

Indium phosphide quantum dots (InP QDs) are nontoxic nanomaterials with potential applications in photocatalytic and optoelectronic fields. Post-synthetic treatments of InP QDs are known to be essential for improving their photoluminescence quantum efficiencies (PLQEs) and device performances, but the mechanisms remain poorly understood. Herein, by applying ultrafast transient absorption and photoluminescence spectroscopies, we systematically investigate the dynamics of photogenerated carriers in InP QDs and how they are affected by two common passivation methods: HF treatment and the growth of a heterostructure shell (ZnS in this study). The HF treatment is found to improve the PLQE up to 16–20% by removing an intrinsic fast hole trapping channel (*τ*_h,non_ = 3.4 ± 1 ns) in the untreated InP QDs while having little effect on the band-edge electron decay dynamics (*τ*_e_ = 26–32 ns). The growth of the ZnS shell, on the other hand, is shown to improve the PLQE up to 35–40% by passivating both electron and hole traps in InP QDs, resulting in both a long-lived band-edge electron (*τ*_e_ > 120 ns) and slower hole trapping lifetime (*τ*_h,non_ > 45 ns). Furthermore, both the untreated and the HF-treated InP QDs have short biexciton lifetimes (*τ*_xx_ ∼ 1.2 ± 0.2 ps). The growth of an ultra-thin ZnS shell (∼0.2 nm), on the other hand, can significantly extend the biexciton lifetime of InP QDs to 20 ± 2 ps, making it a passivation scheme that can improve both the single and multiple exciton lifetimes. Based on these results, we discuss the possible trap-assisted Auger processes in InP QDs, highlighting the particular importance of trap passivation for reducing the Auger recombination loss in InP QDs.

## Abstract 2

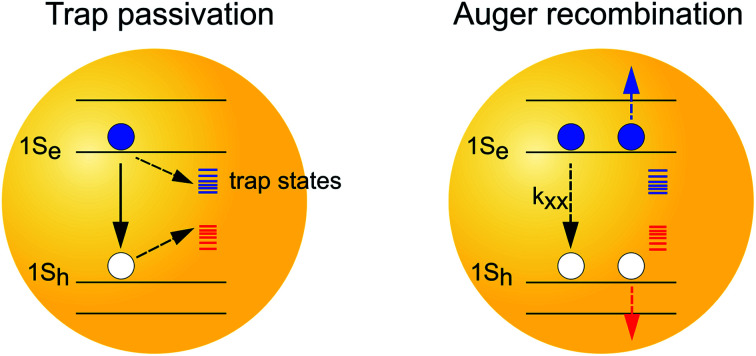
Combined optical spectroscopic study now reveals the photophysical changes of InP QDs upon surface passivation by various methods.

## Introduction

Colloidal quantum-confined nanocrystals, especially spherical quantum dots (QDs), can exhibit a broad tunability of their band-gaps, multiexciton lifetimes and band-edge positions by simply varying the particle sizes,^1^,^2^ enabling their applications in lasing,^3^,^4^ light-emitting diodes (LEDs),^5^–^7^ and solar fuel generation.^8^–^11^ In the past few decades, Cd and Pb-based chalcogenide nanocrystals (*e.g.*, CdSe^3^,^4^ and PbSe^12^) have been widely investigated, leading to significant advancement of our fundamental understanding of exciton and carrier dynamics of QDs and how these properties can be optimized through size, shape and composition control of QDs and heterostructures to improve their device performances.^2^,^13^,^14^ However, the toxic heavy metals in Cd- and Pb-based QDs pose potential human health risks, hindering their commercial applications. Meanwhile, InP, a binary III–V semiconductor, is considered as one of the most promising environmentally friendly nanocrystals. With a bulk bandgap at 1.35 eV and suitable bandedge energies (CB: –3.85 eV and VB: –5.2 eV),^15^ InP QDs can be tuned to absorb a wide range of photoenergies and see emerging applications in solar cells,^16^,^17^ LEDs,^7^,^18^ and photocatalytic reactions.^8^,^10^

**Scheme 1:**
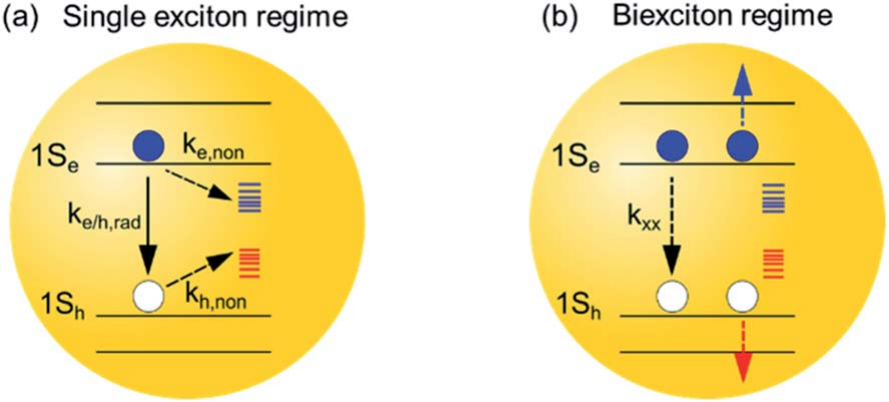
Schematic of photophysical processes in InP QDs in single and multiple exciton states. (a) Single exciton regime: the photogenerated excitons decay predominately through radiative electron and hole recombination (*k*_e/h,rad_), electron trapping (*k*_e,non_) and hole trapping (*k*_h,non_) processes. (b) Biexciton regime: the photogenerated excitons decay through an additional fast Auger recombination process, where the nonradiative decay of one exciton (*k*_xx_) simultaneously promotes another electron (blue line) or hole (red line) into its higher energetic state. In this study, we discuss how surface treatments affect both processes in the InP QDs.

However, despite the desirable material properties, early studies^19^,^20^ have shown that these materials contain a large density of traps states (Scheme 1a), evidenced by their low photoluminescence (PL) quantum efficiencies. Two post-synthetic methods have been developed to passivate the trap states in InP QDs and improve their PLQEs.^20^–^26^ The first passivation method employed a post-synthetic HF treatment of InP QDs under illumination.^20^–^23^,^25^ The HF treatment has been suggested to remove the surface P dangling bonds through a photochemical reaction of trapped holes with the P atom, which is then attacked by the F^–^ ions and eventually detach from the InP surface.^25^ Meanwhile, a recent study suggested an alternative/complementary mechanism where the F^–^ treatment may act through removing the electron traps caused by the surface indium dangling bonds.^22^ Thus, the underlying mechanism remains to be further clarified. The second passivation method, also a general method for many other nanocrystals, is to grow an inorganic shell around the InP core.^18^,^24^,^27^ Although the epitaxial growth of shells around a nanocrystal core is known to passivate the surface states of the core materials,^28^–^31^ it is unclear whether electron and/or hole traps are removed and how this method differs from the HF treatment. Therefore, a clear understanding of these two passivation methods and their impact on exciton and carrier dynamics can provide important insights towards the rational design and improvement of InP QDs for many applications.

Furthermore, many optoelectronic applications of QDs, *e.g.*, lasing^3^,^4^ and LED,^5^–^7^ involve multiexciton states under operational conditions. The dominating energy loss mechanism in the multiexciton regime is through Auger recombination (AR) processes,^32^–^34^ where the nonradiative decay of one exciton simultaneously promotes another exciton or carriers into its higher energetic state (Scheme 1b). As such, the study of AR processes in QDs, especially exploring effective ways to suppress the AR processes in QDs, is crucial for the development of optoelectronic devices with improved performance.^32^,^33^,^35^,^36^ So far, the impact of surface passivation on multiexciton states in InP QDs remains poorly characterized and understood.^37^ It is important to develop surface passivation schemes that can improve the lifetime of not only the single but also the multiple exciton states.

Herein, we examine the mechanism by which the HF treatment and core/shell structure improve the PLQE of InP QDs and the effect of these treatments on single/multiple exciton lifetimes. We directly measure how these passivation schemes affect the electron and hole trapping processes by comparing transient absorption and time-resolved photoluminescence decay kinetics. We show that HF treatment predominately removes hole traps, while the growth of the ZnS shell can effectively remove both electron and hole traps on InP QDs. More interestingly, we observe that the biexciton lifetime of InP QDs is significantly shorter than CdSe QDs of similar sizes. While HF treatment has a minor impact on this short biexciton lifetime, the growth of a ZnS shell (∼0.2 nm) around the InP core can result in a dramatic 20-fold increase of the biexciton lifetime in InP QDs. The latter effect has not been reported previously for Cd chalcogenide QDs. These results highlight that, as compared to traditional Cd-chalcogenide nanocrystals, rational surface treatment is more crucial for InP QDs to passivate trap states and extending biexciton lifetimes for various optoelectronic applications.

## Results and discussion

### Existence of traps states in InP

**Fig. 1:**
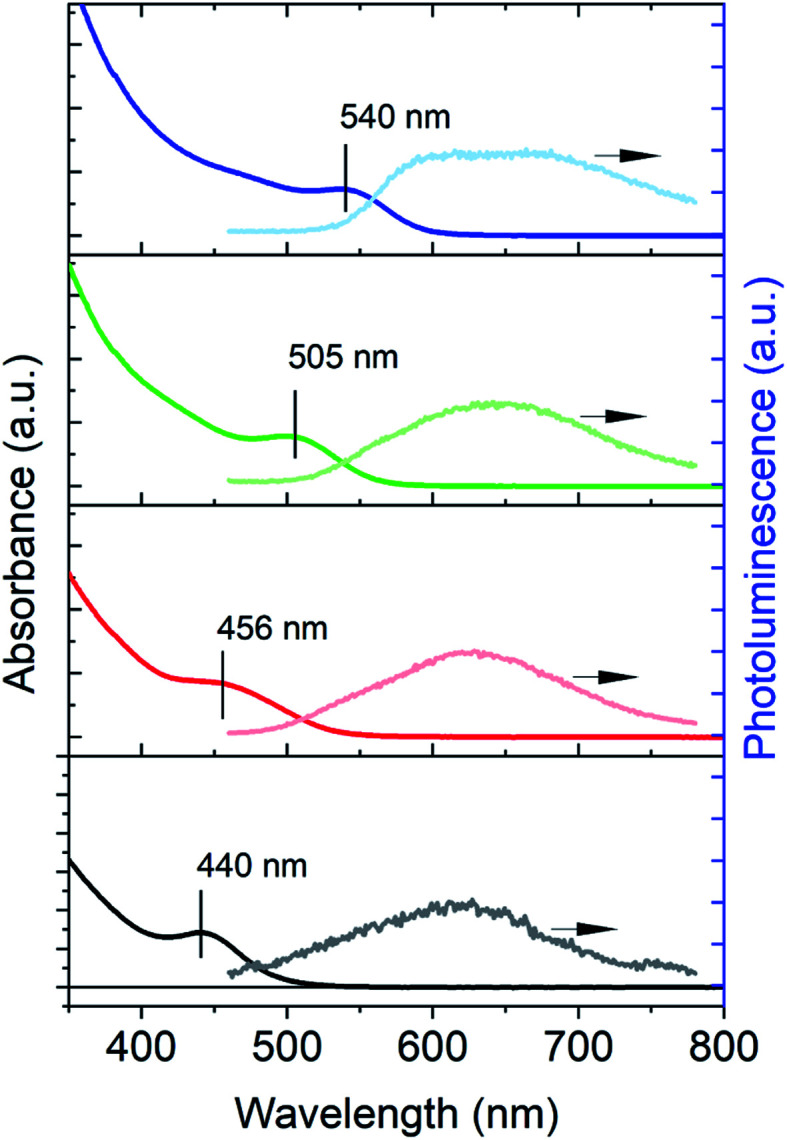
UV-vis absorption (left axis) and photoluminescence (right axis) spectra of InP QDs of different sizes. The peak positions of the band-edge exciton absorption band are indicated in the spectra. The PL spectra are dominated by broad trap state emission.

The InP QDs used in the present study were synthesized by following a recently developed “greener” procedure using tris(diethylamino)phosphine as the phosphine precursor in the present of Zn^2+^ additives.^8^,^27^,^38^ Fig. 1 shows the UV-vis absorption and PL spectra of the as-synthesized InP QDs of four different sizes (estimated diameters: ∼2–3 nm (ref. 39)) with band-edge exciton absorption peaks at 440, 456, 505 and 540 nm, respectively. The PL spectra of these QDs show only weak, even negligible, emission from the band-edge exciton and are dominated by broad emission at the longer wavelength (between 600–800 nm). The broad PL emission in InP QDs has also been previously observed for InP synthesized from other methods and attributed to the hole trap-assisted emission,^15^ similar to those reported in Cd-based nanocrystals.^40^ The absence of band-edge exciton emission and the appearance of the trap-assisted emission indicates fast carrier trapping by trap states in the as-synthesized InP QDs. As such, the PLQEs of all as-synthesized InP QDs are low (<1%), which highlights the importance of understanding the trap passivation mechanisms in InP QDs. Below we use InP QDs with the band-edge exciton absorption at 525 nm as a model system to investigate the exciton dynamics of untreated InP QDs and their passivation mechanisms by both HF treatment (or “HF etching”^20^) and ZnS shell growth.

### Exciton dynamics and carrier trapping processes in untreated InP QDs

**Fig. 2:**
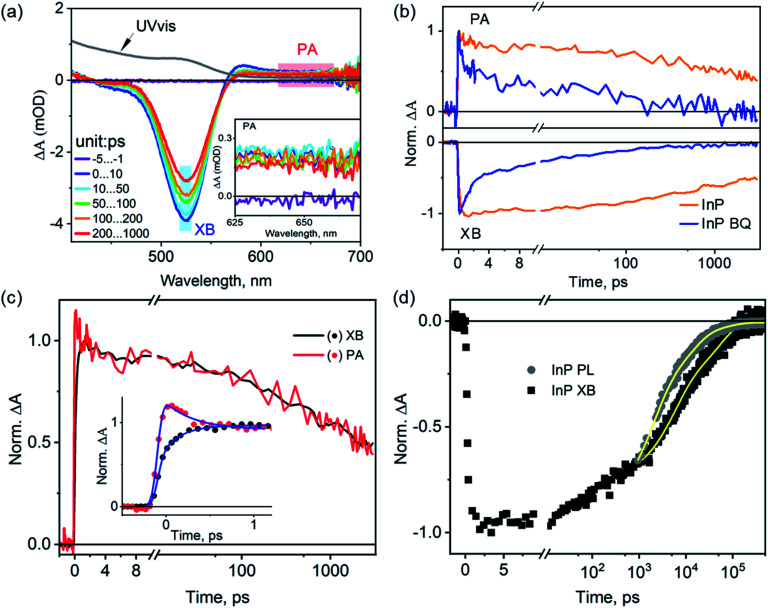
Transient absorption (TA) spectra and exciton dynamics of as-synthesized InP QDs. (a) TA spectra of InP QDs at different time delays after photoexcitation at 400 nm. Also shown for comparison is the UV-vis absorption spectrum of the sample (grey line). The shaded areas mark the exciton bleach (XB) and photoinduced absorption (PA) regions that are averaged and used for kinetic analysis. Inset: enlarged view of the TA spectra in the PA region between 625 and 700 nm. (b) Comparison of XB (bottom) and PA (top) decay kinetics of free InP QDs and QDs in the presence of benzoquinone (BQ) as the electron acceptors. (c) Comparison of XB and PA decay kinetics in InP QDs. The inset shows the expanded view of early time kinetics and fits (solid lines). (d) Comparison of the PL decay of the band-edge exciton (grey dots) monitored at 550 nm with the TA XB decay (black dots). These traces were normalized to have the same amplitude at 1 ns. Solid lines are fits to empirical three exponential decay functions.

We start with the assignment of spectral features in the transient absorption (TA) spectra of untreated InP QDs and then use these features to characterize their exciton dynamics. Fig. 2a shows the TA spectra of InP QDs after photoexcitation at 400 nm under low fluence, corresponding to an estimated average exciton number per QD of ∼0.07 (Fig. S1†). Thus, the following results represent the exciton dynamics of InP QDs in the single exciton regime. The TA spectra show two major features: a strong bleach region between 450 nm and 550 nm (labeled XB below) and a weak photoinduced absorption region from 625 to 700 nm (labeled PA below, Fig. 2a inset). In QDs, the XB results from the state-filling effect of photogenerated electrons and/or holes, which block the ground-state excitonic transition of the QDs and leads to an absorbance decrease (or bleach) feature in TA spectra.^2^,^41^,^42^ Due to the large difference in the effective mass of electrons (*m*_e_ = 0.08) and holes (*m*_h_ = 0.64) in InP QDs,^15^ the density of hole states is much larger than that of electrons, and XB signal is expected to originate mostly from the state-filling effect of electrons. The nature of PAs in QDs vary between different systems and have been previously shown to originate from the intraband transition of photogenerated electrons^15^ or free/trapped holes.^40^,^43^ To verify the spectral origins of the XB and PA regions, we conduct TA measurements with the selective addition of electron scavengers, benzoquinone (BQ), into the QD sample. The addition of electron scavengers can induce a corresponding electron transfer from the InP QDs to the acceptor molecules, thus reducing the amplitude of TA signals originating from electrons. Fig. 2b shows that the addition of BQ into InP QDs results in a faster decay for both the XB and PA regions, with almost complete quenching achieved in <1 ns (∼97%, original TA spectra shown in Fig. S2†). Similar fast decays of XB and PL signals have been observed previously for InP QD/methylviologen complexes and were attributed to electron transfer from the QD to the electron acceptor.^15^ Thus, the above results, strong quenching of both XB bleach and PA signals in the presence of electron scavengers and the large difference in electron and hole effective mass, indicate that both the XB and PA in the TA spectra of InP QDs originate predominantly from the photogenerated electrons. This assignment is further supported by the identical decay kinetics of the XB and PA signals in the absence of any scavengers (Fig. 2c). It should be noted that the early-time PA decay (within 1 ps) is, however, not identical to that of the XB decay. There exists a simultaneous decay of the PA signals (0.22 ± 0.05 ps) and an increase of the XB signals (0.32 ± 0.05 ps) (Fig. 2c inset). This early-time mismatch is attributed to the spectral extension of the PA signals into the XB region, which causes the decay of the PA signals to manifest themselves as a rise in the XB region. Similar spectral overlap and their assignments in InP QDs have also been previously reported,^15^ indicating that the spectral natures of the XB and PA in InP QDs are not sensitive to their synthesis methods.

With the above spectral assignment, we now focus on understanding the exciton decay dynamics of InP QDs. Due to the existence of trap states,^20^,^22^,^23^,^44^ the XB decay in InP QDs should reflect the decay of photogenerated electrons through both the radiative channel by the electron–hole recombination process (*k*_e/h,rad_) and the nonradiative channel by the electron trapping process (*k*_e,non_) (Scheme 1a). Meanwhile, the PL decay of the band-edge exciton (*k*_PL_) reflects the radiative and nonradiative decay from both electrons and holes. Therefore, by directly comparing the PL decay of the band-edge exciton with the XB decay from the TA measurement^23^,^45^ (Fig. 2d), one can calculate the nonradiative decay component of holes (*k*_h,non_, Scheme 1a) through eqn (1).

1 *k*_PL_ = *k*_e/h,rad_ + *k*_e,non_ + *k*_h,non_

Fig. 2d shows the PL decay of InP QDs monitored at 550 nm, where the emission is predominantly from the band-edge exciton (Fig. 1 and more discussions in Fig. S3†). Due to the ns resolution of our setup (instrument response time, IRF ∼ 0.5 ns), we compare the PL and XB decay by normalizing them at 1 ns. As such, the extracted hole dynamics reflect only those longer than ∼1 ns. As shown in Fig. 2d, the PL decay of the InP QDs is found to be much faster than its TA XB decay, indicating the existence of hole trapping processes in addition to radiative electron–hole recombination and electron trapping. From the best fit to an empirical three-exponential decay function, the amplitude-weighted average time constants of PL and XB decays are calculated to be ∼3.1 and 26.7 ns, respectively (detailed parameters listed in Table S1†). Using eqn (1), the time constant of the hole-trapping processes can be therefore estimated to be ∼3.4 ns, which is significantly faster than the radiative decay time (>26.7 ns). This fast trapping time constant is consistent with the observation of weak band-edge exciton emission and strong trapped hole-assisted emission shown in Fig. 2b. In the following section, we will illustrate how the two passivation methods for InP QDs: the HF treatment and the growth of a ZnS shell, affect the exciton decay and carrier trapping processes in InP QDs.

### Exciton dynamics in HF treated InP QDs: passivation of hole traps

**Fig. 3:**
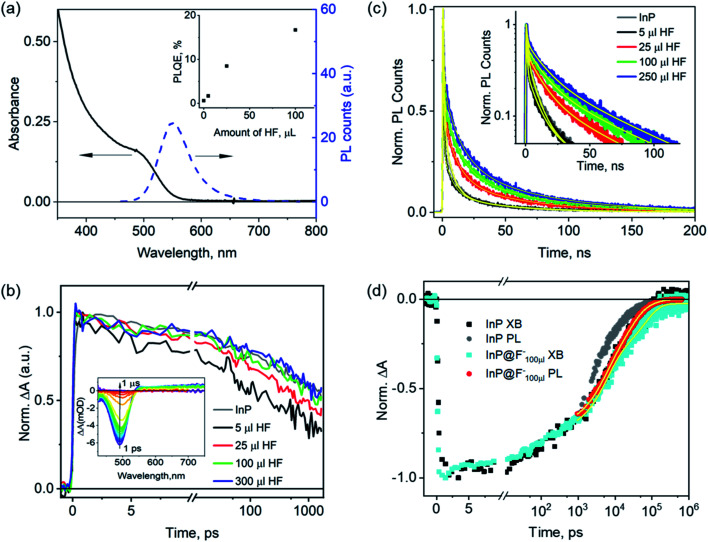
Impact of HF treatments on the carrier lifetime of InP QDs. (a) UV-vis absorption and PL emission spectra of InP QDs after the treatment with 100 μl HF(InP@F^–^_100 μl_). Inset: dependence of PLQE of InP@F^–^ QDs on the HF amount used during the treatment. (b) Change of the XB decay of InP@F^–^ QDs as a function of the HF amount used in the treatment. Inset: TAS of InP@F^–^_100 μl_ QDs. (c) Change of the PL decay of InP@F^–^ QDs as a function of the HF amount used in the treatment and their multiexponential fittings (yellow lines). Inset: same data with *Y*-axis in a logarithmic scale. (d) Comparison of the PL and XB decays of InP and InP@F^–^_100 μl_ QDs. The results for InP QDs are taken from Fig. 2d.

The HF treated InP QDs (named as InP@F^–^ QDs) were prepared by adding different amounts of diluted HF solution (0.527 ml HF 45 wt%, 0.0625 ml H_2_O and 5 ml butanol) into InP QDs solution and illuminating (xenon lamp, 10 mW cm^–2^) the mixtures under ambient conditions, following literature procedures.^20^ The HF amounts were varied from 5, 25, 100, to 250 μl, resulting in the estimated HF/InP molar ratios of 480, 2410, 9630, 24 100, respectively, and these samples are referred to as InP@F^–^_5 μl_, InP@F^–^_25 μl_, InP@F^–^_100 μl_, InP@F^–^_250 μl_ below. Fig. 3a shows the absorption and photoluminescence spectra of InP@F^–^_100 μl_ as an example to illustrate the spectral changes after the HF treatment; data for other samples are similar and shown in Fig. S4 and S5.† After the treatment, the band-edge absorption peak of the InP QDs is blue-shifted from 525 to 485 nm; the PL spectra change significantly with the appearance of an intense band-edge emission peaked at 550 nm and the almost complete suppression of the hole trap-assisted emission. Furthermore, the PLQE of the InP@F^–^ QDs is found to improve gradually with the increase in the HF amounts (inset in Fig. 3a), with the highest PLQE of 16–20% achieved in InP@F^–^_100 μl_. Further increase of the HF concentration results in, however, the aggregation of the InP@F^–^ QDs (Fig. S6†), where the strong light scattering nature of the sample hinders a precise quantification of PLQE and is thus omitted from the discussion below. The observed improvement of InP PLQE by HF treatment is in good agreement with previous reports.^20^–^22^,^25^ Below we investigate the effect of HF treatment on carrier dynamics and the mechanism of PLQE improvement of InP@F^–^ QDs by time-resolved spectroscopy.

The inset in Fig. 3b shows the TA spectra of InP@F^–^_100 μl_ after 400 nm photoexcitation. In comparison with the TA spectra of the untreated InP QDs (Fig. 2a), the XB peak is blue-shifted to 485 nm, in agreement with the shift in their ground-state absorption spectra (Fig. 3a); data for other samples are similar and shown in Fig. S7.† Comparison of the normalized XB decay of samples with different HF treatment (Fig. 3b) shows that the XB decay of the InP@F^–^ samples is faster than the untreated InP QDs (black line in Fig. 3b) at low HF concentration (InP@F^–^_5 μl_ and InP@F^–^_25 μl_); and is the same as the untreated InP QDs at higher HF concentration. Control measurements show that in the absence of HF, illumination of the InP QDs results in the largest acceleration of the XB decay (Fig. S8†). Thus, we attribute this faster XB decay at low HF concentration to the photodegradation of InP, probably due to the formation of an oxide layer around InP QDs^46^ which may act as an additional electron trap. With enough amounts of HF (InP@F^–^_100 μl_ and InP@F^–^_300 μl_), this degradation pathway is, however, effectively suppressed, resulting in identical decay dynamics of XB in InP@F^–^ QDs as the untreated InP QDs.

The comparison of the normalized PL decay of the band-edge exciton at 550 nm for InP@F^–^ QDs (Fig. 3c) shows that the PL decay lifetimes of all samples are longer than the untreated InP QDs, and the lifetime increases at higher concentrations of HF treatment. Because the electron decay of InP@F^–^ is either faster or remains unchanged compared to untreated InP QDs (see above), the prolonged PL decay must therefore arise from a slower hole trapping rate caused by the HF treatments according to eqn (1). In other words, HF treatments can effectively remove the hole traps in InP QDs. To quantify the change of hole trapping rate, Fig. 3d compares the XB and PL decay of the best InP@F^–^ sample, InP@F^–^_100 μl_, using a similar approach as that in Fig. 2d. The XB (black line) and PL decay (grey line) of the untreated InP QDs is also plotted for comparison. Fitting of the XB and PL decays of InP@F^–^_100 μl_ by multi-exponential functions results in the amplitude weighted-average time constants of ∼32.4 and 18.1 ns, respectively (Table S2†), from which the hole trapping time constant in the InP@F^–^_100 μl_ sample is estimated to be ∼41.0 ns (eqn (1)). A previous X-ray photoelectron spectroscopic study of InP QDs suggests that the HF treatment proceeds through the removal of P dangling bonds by F^–^ under illumination,^25^ in agreement with this report, our optical spectroscopic study result shows that HF treatment also reduces hole trap densities in InP QDs synthesized herein^8^,^27^,^38^ and slows down the hole trapping time constant from ∼3.4 to 41.0 ns. Our result also agrees with recent calculations, which suggested that the presence of the F^–^ termination on the InP surface decreases the oscillator strength of its excitonic transition, thus resulting in longer radiative PL decay.^22^ On the other hand, our result is not consistent with other reports that suggest that HF treatment removes electron traps on InP QDs by the passivation of surface indium dangling bonds.^22^,^23^

### Exciton dynamics in InP@ZnS core@shell QDs: passivation of both electron and hole traps

**Fig. 4:**
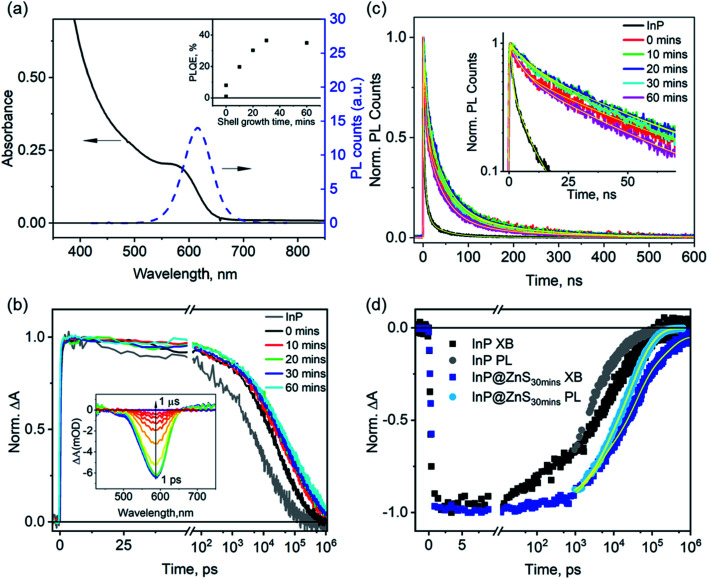
Impact of the ZnS shell growth on the carrier lifetime of InP@ZnS QDs. (a) UV-vis absorption and PL spectra of InP/ZnS after 30 min ZnS shell growth. Inset: dependence of the PLQE of InP@ZnS QDs on the ZnS shell growth time. (b) XB decays of InP@ZnS QDs as a function of the ZnS shell growth time. Inset: TA spectra of InP@ZnS after 30 min of ZnS shell growth. (c) PL decays of InP@ZnS QDs as a function of the ZnS shell growth time and their multiexponential fits (yellow lines). Inset: same data with the *Y*-axis plotted in a logarithmic scale. (d) Comparison of the PL and XB decays of InP@ZnS QDs after 30 min of ZnS shell growth, the sample with the highest PLQE (∼35–40%) in this study. Also shown for comparison are the XB (black dots) and PL (grey dots) decays of untreated InP QDs (data taken from Fig. 2d).

The ZnS shell growth is achieved by the gradual injection of a 2 M sulfur solution dissolved in trioctylphosphine into the as-synthesized InP QD solution at 260 °C.^8^ To understand the effects of the ZnS shell on the carrier dynamics, we sampled the solution during the ZnS shell growth at different times: 0 (right after the injection of all the S precursor), 10, 20, 30, and 60 min, and measured the optical properties of these aliquots (named below as InP@ZnS_0 min_, InP@ZnS_10 min_, InP@ZnS_20 min_, InP@ZnS_30 min_, respectively). Fig. 4a shows the absorption and PL spectra of the InP@ZnS_30 min_ sample as an example to demonstrate the spectral changes of InP QDs due to the ZnS shell; data for other InP@ZnS samples are similar and shown in Fig. S9 and S10.† After coating the ZnS shell, the band-edge exciton absorption peak of InP@ZnS core@shell QDs is red-shifted from 550 nm (peak of the InP core) to 590 nm; the PL of the QDs is significantly improved, with the appearance of a well-defined band-edge emission peak at 620 nm (full width half maximum: ∼60 nm). Meanwhile, the PLQE of the InP@ZnS samples increases with the shell growth time until stabilizes at 30 min (inset in Fig. 4a), with the maximum achievable PLQE of ∼35–40%, similar to previous results based on a similar synthesis approach.^8^,^27^ For InP@ZnS_30 min_, the thickness of the shell is estimated to be ∼0.2 nm by combining both the TEM measurement (Fig. S11 and 12†) and the effective mass approximation (EMA) calculations (ESI Note S2f†). Due to the large band-edge energy offset between InP (CB: –3.85 eV, VB: –5.2 eV)^15^ and ZnS (CB: –3.1 eV, VB: –6.6 eV),^47^ the core@shell InP@ZnS QDs studied here is expected to form a type-I structure, *i.e.*, both the electron and hole wavefunctions are mostly confined within the InP core with only small tunneling into the ZnS shell. As such, their absorption spectra are expected to show a small redshift compared to InP core only QDs. The observed absorption spectra of InP@ZnS QDs are in good agreement with the expected type I band assignment and the calculated spectra by the EMA method (Fig. S2e-1†).

To understand the passivation mechanism of InP QDs by the ZnS shell, we compare the XB and PL decays of the InP@ZnS QDs sampled during the shell growth at different times. The TA spectra of InP@ZnS_30 min_ after 400 nm photoexcitation (the inset in Fig. 4b) show a XB feature centered at ∼590 nm, consistent with its ground state absorption; data for other samples are similar and shown in Fig. S13.† The average time constants of the XB decay increase immediately from ∼26.7 ns in untreated InP to 70.0 ns in InP@ZnS_0 min_ and then more gradually to 156.7 ns in InP@ZnS_60 min_ with the further growth of the ZnS shell (Fig. 4b, Table S3†), indicating a dramatic effect of shell growth on the conduction band electron lifetime. Due to the above-mentioned type-I confinement structure of the InP@ZnS QDs and the small thickness of ZnS shell, the growth of ZnS shell should only have little impact on the electron–hole radiative recombination process. In the ESI Note S2e,† we estimate, using EMA, that this change should be about 3% and cannot account for the large change of the electron lifetimes observed here. Instead, the above results indicate that the ZnS shell, in contrast with HF treatments, can lead to effective passivation of electron trap states in InP@ZnS QDs and extend the electron lifetime.

Unlike the XB decays, the PL decays of these InP@ZnS samples do not show monotonic dependence on the growth time of the ZnS shell (Fig. 4c). These PL decays can be well fitted to three exponential functions. The amplitude-weighted average PL lifetime (Table S4†) increases immediately from 3.0 ± 0.0 ns for InP to 45.5 ± 0.3 ns for InP@ZnS_0 min_ after the addition of S at 0 minute; continues to lengthen for InP@ZnS_10 min_ (49.4 ± 0.2 ns) and InP@ZnS_20 min_ (50.6 ± 0.3 ns) with the growth of the shell; and finally decrease slightly for InP@ZnS_30 min_ (46.3 ± 0.2 ns) and InP@ZnS_60 min_ (37.3 ± 0.0 ns) with the further growth of the shell. Using eqn (1), we calculate the hole decay time constants to be 129.9, 89.3, 83.4, 73.8 and 48.9 ns for InP@ZnS_0 min_, InP@ZnS_10 min_, InP@ZnS_20 min_, InP@ZnS_30 min_, InP@ZnS_60 min_, respectively. These hole trapping time constants are all significantly slower than that (∼3.4 ns) in the untreated InP QDs (Fig. 2d), therefore indicating that the growth of the ZnS shell also passivates the hole traps. However, increasing the ZnS shell growth appears to increase the hole trapping rates with the trapping time decreasing from 129.9 to 48.9 ns. Although shell growth has been shown to induce lattice constrain and increase hole trapping time, the thickness of shells are relatively small in our sample (∼0.2 nm in InP@ZnS_30 min_) and is unlikely to cause the observed PL changes.^48^,^49^ Recent studies suggested that the growth of the ZnS shell around InP QDs can introduce interior/surface lattice disorder due to the incorporation of Zn^2+^ into the InP lattice,^44^,^50^ which introduces new trap states with broad PL emission.^44^ We therefore tentatively attribute the observed faster hole trapping rate at long shell growth time to the incorporation of Zn^2+^ into the InP lattice. As such, although the prolonged growth of ZnS can further improve the electron lifetime, it also leads to the shortening of the hole lifetime; as a result of these two competing effects, the PLQE levels off after 30 minutes of shell growth time (Fig. 4b inset). These above results demonstrate that the growth of a thin ZnS layer can effectively passivate both the electron and hole traps in InP QDs, unlike the HF treatment that only passivates hole traps herein. Our results also show that the initial addition of S precursor has the most pronounced effects on the passivation of both electron and hole traps. Similarly, recent study^23^ has shown that post-synthetic treatment of InP QD with Cd^2+^ or Zn^2+^ cations, which are thought to replace the In^3+^ on its surface, can result in the improvement of the PLQE approximately from 1 to 50%. Although the exact chemical nature of these surface treatments remains to be further understood, these results collectively demonstrate that the surface of InP QDs is indeed the key to the improvement of their PLQE and optoelectronic applications.

### Biexciton Auger recombination processes in InP, InP@F^–^ and InP@ZnS QDs

**Fig. 5:**
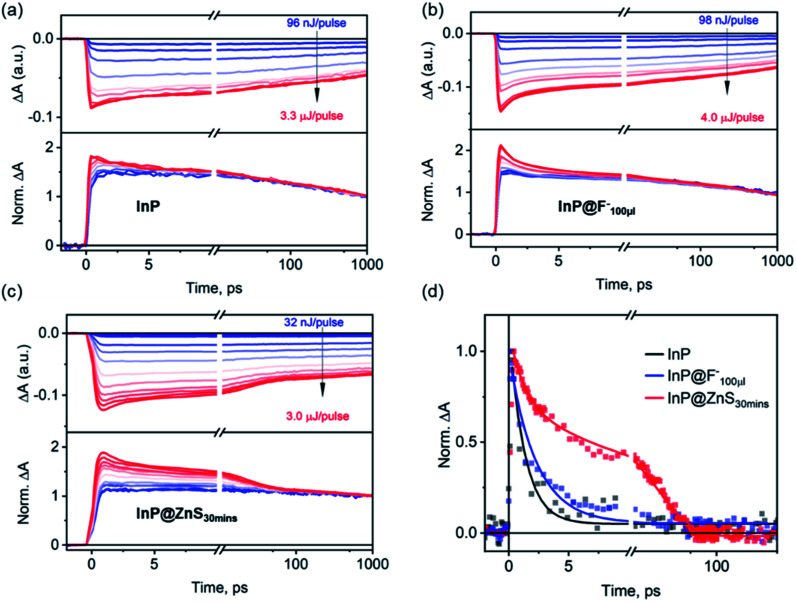
Biexciton Auger recombination processes in InP, InP@F^–^_100μl_ and InP@ZnS_30 min_ QDs. The XB decay kinetics of (a)InP, (b) InP@F^–^_100μl_ and (c) InP@ZnS_30 min_ QDs as a function of excitation fluences. Upper panels show the original fluence dependent traces. Lower panels show the kinetics of all traces normalized at 1 ns. (d) Comparison of the fast decay components (dots) in the normalized decays of (a)–(c) and their fits by exponential decay functions (solid lines). The fast component is extracted by subtracting the highest fluence decay kinetics by the lowest fluence decay kinetics, which represents the biexciton Auger recombination processes in the corresponding samples (see main text for details).

Finally, we compare biexciton decays in InP, InP@F^–^ and InP@ZnS QDs and discuss the effect of HF treatment and ZnS shell growth on biexciton lifetimes. To study this, we follow previous procedures^51^,^52^ by monitoring the XB decay kinetics of QDs as a function of the excitation fluences as shown in Fig. 5a–c for InP, InP@F^–^_100μl_ and InP@ZnS_30 min_ QDs, respectively. The corresponding fluence dependent TA spectra are shown in Fig. S14–16.† In these kinetic analyses, the contribution from the overlapping PA signal, as mentioned above (Fig. 2c), has been subtracted. In all cases, at low excitation fluence, the XB decay is slow (>1 ns), consistent with the fact that the TA signal is dominated by QDs with one exciton. At higher excitation fluences, as the averaged exciton number per QDs increases, both the amplitude of the total XB signal and the amplitude of a fast decay component increase. The appearance of the latter can be better seen in the lower panels of Fig. 5a–c, where the decay kinetics at different excitation fluence has been normalized at 1 ns. These normalized decay curves show identical decays starting from 100–1000 ps, indicating that after ∼100 ps, the XB signal reflects the decay of single exciton states (Scheme 1a). The normalized XB kinetics also show a fast decay channel within 1–100 ps whose amplitudes increase at higher fluences, and the decay time constants are independent of the fluence. This fast component can be attributed to the Auger recombination (AR) of multiple excitons. Because the XB decay in InP QDs reflects only the dynamics of the band-edge 1S_e_ electrons with a two-fold degeneracy,^53^ the faster component is a direct probe of the biexciton AR process (Scheme 1b),^54^,^55^ whose contribution increases as the percent of excited QDs with *n* = 2 states increases at higher fluences. Although at higher fluence, the contribution of *n* > 2 exciton states also increase, their effect cannot be directly probed at the 1S XB which saturates at *n* = 2.^56^,^57^ To obtain the biexciton decay kinetics, we subtract the XB decay of each sample under the highest fluence ( To obtain the biexciton decay kinetics, we subtract the XB decay of each sample under the highest fluence (〈*N*〉 estimated to be 1.0, 1.5, 1.5 for the InP, InP@F estimated to be 1.0, 1.5, 1.5 for the InP, InP@F^–^_100μl_ and InP@ZnS_30 min_, respectively, Fig. S17†) by its decay under the lowest fluence, and the resulting biexciton decays kinetics are shown in Fig. 5d. For InP and InP@F^–^_100μl_ QDs, the decay kinetics can be well fit by a single exponential decay function to reveal a biexciton lifetime of InP and InP@F^–^_100μl_ of 1.3 ± 0.1 and 2.3 ± 0.2 ps, respectively. For InP@ZnS QDs, a satisfactory fit to the decay kinetics requires a biexponential function with time constants (amplitudes) of 1.3 ± 0.2 (∼36 ± 2%) and 20.2 ± 1 (∼64 ± 2%) ps, respectively. The faster component is similar to the biexciton lifetime of the untreated InP QDs characterized above. We attribute this to the non-uniform ZnS shell growth around InP QDs, which produces a portion of InP QDs without ZnS shell. This nonuniformity can be rationalized by considering the irregular shape of InP QDs, evidenced in Fig. S12† and reported previously,^50^ and the very thin thickness of ZnS shell studied here. The slower component (20.2 ± 1 ps) can be therefore assigned to the biexciton Auger lifetime of InP@ZnS QDs. Importantly, these results show that although the HF treatment is efficient in removing the surface hole traps, it is, however, not sufficient to extend the biexciton lifetime of InP QDs used in the current study. The growth of the ZnS shell can, on the other hand, result in a dramatic 20-fold improvement of the biexciton lifetime in InP@ZnS QDs.

The measured biexciton lifetime in InP QDs (1.3 ± 0.2 ps) is considerably faster than previously reported biexciton lifetimes of CdSe QDs (8.5 ps) of a similar size (2.4 nm).^55^ Similarly, other InP QDs used in the present studies (440 nm, 456 nm and 505 nm in Fig. 1) also show ultrafast biexciton lifetime (all <1 ps, Fig. S18†). These Auger lifetimes in the untreated InP QDs also seem faster than expected results (∼10 ps) predicted by the “universal volume scaling law”, where the Auger recombination rate was showed to follow an inverse *R*^3^ dependence regardless the chemical nature of the quantum dots.^58^ Furthermore, the 20-fold improvement in Auger lifetime by a thin type-I ZnS shell is unexpected. Though the growth of ZnS shell can delocalize the electron and hole wavefunctions into the ZnS and therefore reduce the overlap of electron and hole wavefunctions in the Auger process, the type-I nature of the core/shell structure used here should only result in a minor electron and hole delocalization (Fig. S2e-1†). Even for the type-II structure, where the electron and hole wavefunction overlap can be significantly reduced, a previous study has shown that the growth of ∼0.2 nm thick CdS shell around a 2.4 nm core CdSe only resulted in a ∼3.5 fold increase of the biexciton lifetime from 8.5 to 29 ps.^55^ Thus, the underlying mechanism of this significant improvement of biexciton lifetime by the ZnS shell seems to go beyond the simple consideration of the effect of type-I heterostructure on the electron and hole wavefunctions.

The key passivation mechanism difference between the HF and ZnS treatment is the significant electron trap passivation by the growth of ZnS shell. We hypothesize that the large density of electron trap states in InP and InP@F^–^ QDs is likely responsible for their fast Auger recombination. In bulk semiconductor, the trap-assisted Auger processes are known to cause a faster decay of the biexciton states.^59^–^61^ This effect was also recently suggested to occur on ZnO nanocrystals to account for its abnormal volume scaling law and fast Auger process.^62^ Specifically, in n-doped ZnO nanocrystals,^62^ the negative trion state can decay by the recombination between a conduction band electron with a surface trapped hole while exciting the additional electron to a higher energy state. The involvement of a localized trap states may further relax the requirement of momentum conservation and thus accelerate the Auger decay rate.^63^ Thus, it is possible that the effective removal of surface electron trap states by the growth of ZnS shell suppresses the fast trap-assisted Auger decay channel and lengthens the biexciton lifetime in InP@ZnS core/shell QDs. To precisely understand the trap-assisted Auger processes, further high-level atomistic calculation is required and should provide crucial correlation between Auger efficiency and the nature of the trap states. This precise understanding of the nature and location of trap states and its photophysical impact are vital for a deeper understanding and more rational design of InP QDs.

The results reported above reveal different passivation mechanisms by HF treatment and the growth of ZnS shell for the InP QDs synthesized herein. It also reveals the dramatic difference of Auger lifetime of InP QDs as a result of the surface treatments. Because the central role of single and multi-exciton lifetimes in optoelectronic applications, these results provide useful insight for a rational design and improvement of these materials. The further optimization of core/shell heterostructures or a combined application of both HF treatment and the core–shell passivation schemes,^7^ holds the promise for improvement of InP QDs. It is noted that, in the state-of-art high PLQE InP QDs, a fine gradient core–shell structure was utilized to tune the carrier wavefunction delocalization and passivate the surface trap states,^7^,^18^,^49^ achieving near unity PLQE. For these highly emissive InP QDs, the surface trap states are likely effectively passivated to minimize the effect of electron–hole nonradiative recombination. Furthermore, the reduced overlap of the carriers' wavefunctions afforded by the gradient core/shell structure and the reduced trap-assisted Auger process likely have a combined effect in extending their bi- and multi-exciton lifetime, therefore enhancing their performances as light-emitting materials.

## Conclusion

In summary, the present study investigates the mechanisms by which HF treatment and InP@ZnS core/shell heterostructures affect the PL quantum efficiency as well as single exciton and biexciton lifetimes in InP QDs. The transient absorption study of exciton and carrier decay dynamics in untreated InP QDs reveals the existence of both fast electron and hole trapping processes. Surface passivation of InP QDs by both the HF treatment and the growth of a ZnS shell (type-I heterostructure) leads to large increases in the PLQE of InP QDs. However, the mechanism of this improvement by HF treatment is predominantly through removing the fast surface hole trapping channel (*τ*_hole_ = 3.4 ± 1 ns), while the growth of a ZnS shell slows down both electron and hole trapping processes simultaneously. Thus, although both surface modifications are effective in improving PLQE, they have different passivation mechanisms and effects on carrier trapping processes, which is important for the further rational improvement of InP QDs for various photocatalytic and optoelectronic applications.

Furthermore, the biexciton lifetime of untreated InP QDs is only ∼1.2 ps, significantly shorter than that of CdSe QDs of similar sizes, and the HF treatment is insufficient to suppress the fast Auger recombination processes. The growth of a thin ZnS shell (∼0.2 nm, approximately one monolayer ZnS), on the other hand, can extend the biexciton lifetime 20-fold, up to 20.2 ± 1 ps. These results highlight that the untreated InP QDs suffer severe Auger recombination losses, representing a major limitation in their development for LEDs and other devices that involve the participation of multiexciton states in operation. On the other hand, core–shell passivation, even without significantly changing the wavefunction overlap between electrons and holes in the InP core, is a very promising approach for reducing this Auger loss. Although the mechanism of this large decrease in biexciton lifetime remains to be further clarified, our results imply the likely significant role of the trap-state assisted Auger processes in InP QDs. Further computational studies on the relationship between trap state passivation and the Auger recombination rates should provide vital insights.

## Associated content

Additional sample characterization and spectroscopic data.

## Conflicts of interest

The authors declare no competing financial interest.

## Supplementary Material

Supplementary information
