# Supplementary material for: Surface passivation extends single and biexciton lifetimes of InP quantum dots
Source: Chem Sci. 2020 May 18;11(22):5779–89. doi: 10.1039/d0sc01039a (PMC7416692; doi:10.1039/d0sc01039a)
Supplement: Supplementary file 1 [file SC-011-D0SC01039A-s001.pdf]

Electronic Supplementary Material (ESI) for Chemical Science.
This journal is © The Royal Society of Chemistry 2020

Supporting information for:

Surface Passivation Extends Single and Biexciton Lifetimes of InP Quantum Dots

Wenxing Yang*†‡, Yawei Yang†§, Alexey L. Kaledin#, Sheng He†, Tao Jin†, James R. McBride¶, Tianquan Lian*†.

† Department of Chemistry, Emory University, 1515 Dickey Drive Northeast, Atlanta, Georgia 30322, USA

‡ Department of Chemistry − Ångström Laboratory, Physical Chemistry, Uppsala University, SE-75120 Uppsala, Sweden

§Electronic Materials Research Laboratory, Key Laboratory of the Ministry of Education, International Center for Dielectric Research,

Shaanxi Engineering Research Center of Advanced Energy Materials and Devices, School of Electronic Science and Engineering, Xi'an Jiaotong University, Xi'an 710049, Shaanxi, P. R. China

#Cherry L. Emerson Center for Scientific Computation, Emory University, 1515 Dickey Drive, Atlanta, GA 30322

¶Department of Chemistry, The Vanderbilt Institute of Nanoscale Science and Engineering, Vanderbilt University, Nashville TN, 37235, USA

Contents

Supplementary Note 1. Experimental sections 4

S1a. Synthesize of InP and InP@ZnS samples 4

S1b. HF treatment of InP 5

S1c. Absorption and emission measurements 5

S1d. TEM measurements 5

S1e. Transient absorption (TA) spectroscopy 5

S1f. Photoluminescent decay measurements 6

Supplementary Note 2. Calculation details 6

S2a. Estimation of the QDs concentration 6

S2b. Convolution fitting equations 7

S2e. Single-band effective mass calculations of the band energy diagram 8

Figure S3. (a) UV-vis and PL spectra of InP QDs with the first exciton position at 525 nm (b) its PL decay when monitored at different wavelength after photoexcitation at 400 nm. 13

Figure S4. UV-Vis absorption spectra of InP QDs after treating with different amounts of HF 14

Figure S5. The normalized PL spectra of InP QDs after treating with different amounts of HF. Normalization is achieved by dividing the original PL counts by the absorptivity of the samples. 14

Figure S6. The visual appearance of InP QDs solution after treating with different amounts of HF. 15

When increasing the HF concentration above 250 µl, the QDs solution becomes aggregates and more scattering, also reflected in the UV-Vis measurement (Figure S1), and hinder the precise determination of its PLE 15

Figure S7. TA spectra of InP after treated with different amounts of HF. Excitation Wavelength: 400 nm. Excitation Fluence: ~ 160 nJ/pulse. 15

Figure S8. TAS measurement on a 2nd batch of InP QDs after treating with different amounts of HF. 16

Figure S9. UV-Vis spectra of InP QDs as a function of the ZnS shell growth time. Inset: Zoomed-in absorption spectra region near the band edge 16

Figure S10. Normalized PL spectra of InP QDs as a function of the ZnS shell growth time. Normalization is done by dividing the original PL counts by the absorptivity of the samples. Inset: Normalized PL at the peak emission intensity. 16

Figure S11. (a) HAADF-STEM picture of InP@ZnS_{30mins} used in the present study. (b) the size distribution of nanoparticles extracted from Figure S11a. 17

Figure S12. (a)-(f) the EDS mapping of InP@ZnS_{30mins} used in the present study. 17

Figure S13. TA spectra of InP QDs with different ZnS shell growth times. Excitation Wavelength: 400 nm. Excitation Fluence: ~100 nJ/pulse. The spectra shown was averaged from the indicated time regions. 18

Figure S14. TA spectra of InP QDs after photoexcitation at 400 nm with different pump fluences. The spectra shown was averaged from the indicated time regions.1 19

Figure S15. TA spectra of InP@$F^-_{100\mu l}$ QDs after photoexcitation at 400 nm with different pump fluences. The spectra shown was averaged from the indicated time regions. 20

Figure S16. TA spectra of InP@ZnS after photoexcitation at 400 nm with different pump fluences. The spectra shown was averaged from the indicated time regions........ 21

Figure S17. Calculated averaged exciton numbers as a function of excitation fluence for the InP, InP@$F^-_{100\mu l}$ and InP@ZnS30_{mins} samples used in Figure S14–16, respectively. The calculation details are described in Supplementary Note S2c. 21

Figure S18. Biexciton Auger recombination processes of other InP QDs used in the present study. The normalized (at 1 ~ ns) XB decay kinetics of (a) InP_{440nm} (b) InP_{456nm} (c) InP_{505nm} QDs at low and high excitation fluence. The Inset show the corresponding normalized difference ΔA (Norm. diff(ΔA)), which is calculated by diff(ΔA)= $\Delta A_{high,fluence}$- $\Delta A_{low,fluence,}$ and their convoluted exponential fittings. Detailed description of the data processes can be found in Figure 5 of the main text. The original TA spectra and the fluence dependent XB decay kinetics are omitted. 22

Table S1 Fitting results of the XB and PL decays of the intrinsic InP QDs (Figure 2d)........ 22

Table S2 Fitting results of the XB bleach and PL decay of the InP@$F^-_{100\mu l}$ sample (Figure 3d) 22

Table S3 Fitting results of the XB bleach of InP@ZnS with different shell growth time (Figure 4b)........ 23

Table S4 Fitting results of the PL decay of InP@ZnS with different shell growth time (Figure 4c)........ 23

Reference 23

Supplementary Note 1. Experimental sections

S1a. Synthesize of InP and InP@ZnS samples

The InP QDs and InP/ZnS QDs were synthesized using Schlenk line techniques according to previous publication with slight changes.[1]

For InP QDs, 0.5 mmol of indium(III) chloride, *x* mmol of zinc(II) chloride and 2-*x* mmol of zinc(II) iodide were mixed in 5.0 mL of oleylamine (x=2, 1.5, 1 and 0.5 are for QDs with excitonic absorption peaks of 550, 525, 480 and 435 nm, respectively). The reaction mixture was first kept under vacuum at 120 °C for 1 h. The mixture was then heated to 180 °C under nitrogen. After reaching 180 °C, 0.5 mL of tris(diethylamino)phosphine was rapidly injected into the mixture. The system was kept at 180 °C to react for 30 min and finally cooled down to room temperature by an ice water bath.

For InP/ZnS QDs, after the above system was kept at 180 °C for 30 min, it was further heated to 260 °C instead of cooling down, and 1.0 mL of trioctylphosphine (TOP)-S (2 M) solution was added with a rate of 0.2 mL min^{-1} (TOP-S solution was made by dissolving 0.128 g of sulfur in 2.0 mL of TOP by ultrasound). The reaction was stopped after 30 min. Finally, the system was cooled down to room temperature.

For purification of the QDs, 10 mL of ethanol were added into the crude product after reaction to precipitate the QDs. After centrifugation at 5500 rmp for 5 mins, the supernatant was discarded. The precipitated QDs were further purified by dissolution in 10 mL of hexane and subsequent precipitation in 20 mL of ethanol. Finally, the QDs were dissolved in hexane and kept at ambient condition.

One should note that the absorption spectra of the as-synthesize InP was found to gradually blue-shifted with time. The kinetic decay of the samples was found to decay slightly faster than it was freshly made. This slight change was verified not to affect the general conclusion drawn in the present work.

S1b. HF treatment of InP

The HF treatment of InP was conducted according to previous publications.[2] In a typical treatment, 1.5 ml InP (OD ~ 0.72 at 413 nm in hexane) is mixed with 11 ml Butanol and 0.125 g TOPO as the stabilizing ligands. The diluted HF solution is composed of 0.527 ml HF (45 wt%), 0.0625 ml H_2O and 5 ml butanol. **Cautious: HF acid is extremely dangerous and special training and waste treatment protocol is required to handle it.** During the optimization of HF concentration, we test the usage of 5 µl, 25 µl, 100 µl, 250 µl diluted HF solution. After adding the HF solution into the InP solution, the mixture was vigorously stirred under light for 10–16 hr (Xenon lamp, ~10 mW/cm^2). No further optimization of the illumination time was conducted but reported previously.[2] Practically, the 100 µl HF treatment was found to give the best PL performance and stability suitable for the spectroscopic study.

S1c. Absorption and emission measurements

All static PL measurements were performed with FluoroMax-3 Spectrofluorometer of HORIBA Scientific. The photoluminescent efficiency is measured using Rhodamine 6G in ethanol as an internal reference. The excitation wavelength is chosen to be 400 nm for all the samples and the scan range is from 420 to 700 nm. Steady-state UV-Vis spectra were recorded with a spectrometer (Aligent 8453).

S1d. TEM measurements

Solutions of the InP/ZnS nanoparticles were deposited onto ultrathin carbon on lacey carbon coated copper TEM grids (Ted Pella #1824) and baked at 145 ˚C overnight under vacuum. STEM-EDS and HRSTEM imaging was performed on a Tecnai Osiris operating at 200 kV equipped with a SuperX™ quad EDS detection system. STEM-EDS maps were acquired using Bruker Esprit 1.9 software with a beam current on the order of 1 nA.

S1e. Transient absorption (TA) spectroscopy

Femtosecond Transient Absorption. The TA measurements were conducted in a Helios spectrometer (Ultrafast Systems LLC) with pump and probe beams derived from a regenerative amplified Ti: Sapphire laser system (Coherent Legend, 800 nm, 150 fs, 3 mJ/pulse, and 1 kHz repetition rate, or Coherent Astrella, 800 nm, 60 fs, 4 mJ/pulse at 1 kHz repetition rate). The output 800 nm light were split into two parts. One part of the 800 nm pulse passed through a frequency doubling BBO crystal and generated the 400 nm excitation beam. Another part of the 800 nm beams was attenuated and focused into a sapphire window for the generation of the white light continuum, with a wavelength range from 420 nm to 800 nm. In some measurements, the white light was generated with a CaF_2 window. The generated white light was split into both a reference and a probe beam using a beam splitter. The probe beam was focused with an aluminum parabolic reflector onto the sample. The reference and probe beams were focused into a fiber optics-coupled

multichannel spectrometer with complementary metal-oxide-semiconductor (CMOS) sensors and detected at a frequency of 1 kHz. During the measurement, the pump laser beam was chopped at a frequency of 500 Hz. The delay between the pump and probe pulses was controlled by a motorized delay stage. The change in absorbance for the pumped and unpumped samples was calculated. Samples in 1 mm cuvettes were used for all spectroscopy measurements under vigorous stir and ambient condition. The solvent response is measured using the pure solvent solution. The excitation power of all the measurements is varied and indicated in the caption of the figures. Results from the two laser systems were determined to be the same.

Nanosecond TA was performed with the EOS spectrometer (Ultrafast Systems LLC). The pump beam at 400 nm was generated in the same way as in the femtosecond TA experiments. The white light continuum (380-1700 nm, 0.5 ns pulse width, 20 kHz repetition rate) was used and generated by focusing a Nd:YAG laser into a photonic crystal fiber. The delay time between the pump and probe beam was controlled by a digital delay generator (CNT-90, Pendulum Instruments). The probe and reference beams were detected with the same multichannel spectrometers used in femtosecond TA experiments. The IRF of this system was measured to be ~ 280 ps.

S1f. Photoluminescent decay measurements

Time-correlated single photon counting (TCSPC) method was used to measure the TR-PL decay of the solution samples. Samples were hold in a 1 cm quartz cuvette and measured at the right-angle geometry. The output pulses centered at 800 nm (∼100 fs, 80 MHz) from a mode-locked Ti:Sapphire laser (Tsunami oscillator pumped by a 10 W Millennia Pro, Spectra-Physics) were passed through a pulse picker (Conoptics, USA) to reduce the repetition rate by a factor of 55 and then frequency-doubled in a BBO crystal to generate pump pulses at 400 nm and used to excite samples. The emissions from samples were detected by a microchannel-plate photomultiplier tube (Hamamatsu R3809U-51), the output of which was amplified and analyzed by a TCSPC board (Becker & Hickel SPC 600). The IRF of this system was determined to be ~0.5 ns by fitting the convolution of the rising kinetics.

Supplementary Note 2. Calculation details

S2a. Estimation of the QDs concentration

The extinction coefficient of the QDs were estimated according to previous publication. Briefly, the extinction coefficient of InP at 413 nm was taken as an intrinsic properties of InP, which is not affected by the quantum confinement as previously described.[3] Taking the diameter of the InP to be 2.7 nm, we estimated its extinction coefficient to be 1.65×10^5 $M^{-1}cm^{-1}$. The corresponding concentration of InP can then be estimated through the Lambert-Beer law.

S2b. Convolution fitting equations

Gaussian IRF:

$$\frac{1}{\sqrt{2\pi\tau_0}}\exp(-\frac{(t-t_0)^2}{2\tau_0{}^2})$$

Where τ_0 is the width of the gaussian beam. t_0 is the time offset of the beam.

Multiple exponential decay Δ*A(t)*:

$$\Delta A(t)=\sum_i^n A_i\exp(-\frac{t-t_0}{\tau_i})+y_0$$

Where A_i and τ_i represent the corresponding pre-factor and time constant of the single exponential decay component. y_0 is the decay offset.

Convolution fitting equations:

$$\Delta A(t)=\frac{A_n}{2}(1-erf\left(\frac{-(t-t_0)}{\sqrt{2}\tau_0}\right))+\sum_1^3\frac{A_i}{2}\exp(\frac{\tau_0{}^2}{2\tau_i^2}-\frac{t-t_0}{\tau_i})(1-erf(\frac{\tau_0{}^2-\tau_i(t-t_0)}{\sqrt{2}\tau_0\tau_i}))$$

Where A_n is a constant and the first term vanishes when y_0=0.

S2c. Estimation of the number of excitons, <*N*>, in QDs

To estimate the average exciton numbers per QD ,<*N*>, in the TA study, we conduct fluence dependent TA study on the samples and monitor the dependence of the XB amplitude at a long decay time (after the complete decay of multi-exciton states) on the excitation fluence. This method have been previously well-established for nanocrystals, with details described in literatures.[4,5]

According to the model,[4,5] the average initially generated exciton numbers per QD, <*N*>, and the average exciton numbers at long decay time (after the bi-and multi-exciton processes), $<N>_{late}$ can be described by by the following equations, respectively:

$$\langle N\rangle = w\times I \qquad \text{S2c.1}$$

$$\langle N\rangle_{late}\propto A_{late}(I)\propto A\times(1-e^{-(w\times I)}) \quad \text{S2c.2}$$

Where *w* is a characteristic coefficient related to the light absorption of the QDs. *I* represent the incident photon flux. $A_{late}(I)$ is the amplitude of the XB at the long decay time (~1000 ps used here). *A* is a scaling

constant that accounts for the decay of single exciton from initial to the long decay time (both radiative and non-radiative), calculated by dividing the XB amplitude at long decay time by its initial amplitude (~0 ps) under very low excitation fluence. In our InP QDs cases, A is taken to be ~ 0.5.

Here, we first normalized the long-time XB signals of the QDs at the saturating fluence to be 0.5 by a normalization factor. Because at a saturation fluence, all QDs will be initially populated with more than one exciton. After bi- and multi- exciton interactions, these QDs are left essentially with one exciton, which undergo a single exciton decay, with the XB amplitude loss defined to be A. We then normalized the long-time XB bleach signals of QDs at all other fluence by the same normalization factor. These processes can therefore produce the estimated averaged exciton number per QDs at the long-time decay time, $<N>_{late}$ (Figure S1) at different fluence. These $<N>_{late}$ data can then be fitted by equation S2c.1, resulting in a w value of ~0.0019 nJ^{-1}. One can then calculate the $<N>$ at difference fluences by equation S2c.2. For data in the Figure 1, $<N>$ is estimated to be ~0.07.

S2e. Single-band effective mass calculations of the band energy diagram

In the single-band effective mass approximation (EMA) employed here, the electron and hole wave functions and energies are calculated by solving coupled Schrodinger-Poisson equations discretized on a grid using a variable-mass DVR,[6–8]

$$\left\{-\frac{\hbar^2}{2}\nabla\cdot[\frac{1}{m_e^*}\nabla]+V_e(\mathbf{r})+\frac{1}{2}\Phi_e^{\text{ind}}(\mathbf{r})+\Phi_h(\mathbf{r})\right\}1S_e(\mathbf{r})=E_e1S_e(\mathbf{r}) \quad , \quad \text{S2e-1a}$$

$$\nabla\cdot\varepsilon_{\text{r}}\nabla\Phi_e(\mathbf{r})=4\pi\left|1S_e(\mathbf{r})\right|^2, \quad \text{S2e-1b}$$

for the electron in $1S_e$, and a corresponding one for the hole in $1S_h$ orbital

$$\left\{-\frac{\hbar^2}{2}\nabla\cdot[\frac{1}{m_h^*}\nabla]+V_h(\mathbf{r})+\frac{1}{2}\Phi_h^{\text{ind}}(\mathbf{r})+\Phi_e(\mathbf{r})\right\}1S_h(\mathbf{r})=E_h1S_h(\mathbf{r}), \quad \text{S2e-2a}$$

$$\nabla\cdot\varepsilon_{\text{r}}\nabla\Phi_h(\mathbf{r})=-4\pi\left|1S_h(\mathbf{r})\right|^2, \quad \text{S2e-2b}$$

although any pair of orbitals is permitted to be occupied. The parameters entering these equations are described in Table 2e-1. The grid spacing parameter used in the calculations is 2 bohr. The curly brackets in S2e-1a and S2e-2a contain, respectively, the effective mass kinetic energy terms, the band potentials for electron and hole, the electrostatic source potentials due to hole and electron, and the self-induced electron and hole potentials with the proper factor of 1/2.[8,9] The Poisson equations are solved subject to the condition that the inner and outer potentials match at the surface boundary defined by a cutoff radius

$R_{cut}=(x_{cut}^2+y_{cut}^2+z_{cut}^2)^{1/2}$ with $\Psi(x_{cut},y_{cut},z_{cut}) = 0$, where Ψ is particle's wavefunction.[6,7] The cutoff radius was taken to be that of the dot, pure or shelled, plus 1 nm. In other words, the wavefunction was allowed to penetrate 1 nm outside dot's surface. The induced potentials Φ_e^{ind} *and* Φ_h^{ind} are derived from the source potentials Φ_e, Φ_h by integrating the induced surface polarization density[10] over all interfacial (InP/hexane for pure and, InP/ZnS and ZnS/hexane for shelled dots) surfaces *I*,

$$\Phi_e^{\text{ind}}(\mathbf{r}) = \frac{1}{4\pi}\sum_I \frac{\varepsilon_{out}^I - \varepsilon_{in}^I}{\varepsilon_{out}^I} \oint \frac{\vec{\nabla}\Phi_e(\mathbf{r})\big|_{in} \cdot \hat{n}_{out}^{in}(\mathbf{r}_I)}{|\mathbf{r}-\mathbf{r}_I|} dA_I \quad \text{S2e-3a}$$

$$\Phi_h^{\text{ind}}(\mathbf{r}) = \frac{1}{4\pi}\sum_I \frac{\varepsilon_{out}^I - \varepsilon_{in}^I}{\varepsilon_{out}^S} \oint \frac{\vec{\nabla}\Phi_h(\mathbf{r})\big|_{in} \cdot \hat{n}_{out}^{in}(\mathbf{r}_I)}{|\mathbf{r}-\mathbf{r}_I|} dA_I \quad \text{S2e-3b}$$

Table S2e-1. Material parameters used in the EMA calculations

	InP[(a)]	ZnS[(b)]	hexane
m_e*	0.08	0.25	1
m_h*	0.64	1.3	1
ε_r	12.5	8.9	1.88
V_e	-3.85	-3.1	0
V_h	-5.2	-6.6	-8.4

m_e* and m_h* are electron and hole effective masses in units of m_0; ε_r is the dielectric constant; V_e and V_h are conduction and valence band edges, respectively, in eV. (a) taken from Ref[11] (b) taken from Ref[12].

Table S2e-2. Energy levels of electron (CB) and hole (VB) in a single exciton in a 2.7 nm InP dot, and its ZnS shelled version, sorted by symmetry. Unit: eV.

	InP(CB)	InP(VB)	InP@ZnS(CB)	InP@ZnS(VB)
1S	-3.488	-5.130	-3.513	-5.144
1P	-2.315	-5.393	-2.577	-5.380
1D	-0.780	-5.727	-1.492	-5.676
1F	-0.014	-6.112	-0.497	-6.013
1G				-6.333
2S	-0.096	-5.867	-0.303	-5.813
2P		-6.080		-5.991
2D		-6.559		-6.399
2F		-7.587		-7.332
2G				-7.747
3S		-7.098		-6.925

We first examine the effect of a 0.2 nm ZnS shell on the band structure of a 2.7 nm InP. The energy levels of the electron and the hole, -3.488 and 5.130 eV in InP, shift, respectively, to -3.513 and -5.144 eV upon

shelling, cf, Table S2e-2. These band edge shifts are minor compared to the 0.75 and 1.4 eV band offset of the conduction and valence band, respectively. Furthermore, electron-hole spatial overlap, which also measures the intensity of the lowest energy interband UV transition, is calculated to be $|\langle 1S_e|1S_h\rangle|^2$ = 0.847233 and 0.822567 for InP and InP@ZnS respectively, and is only 2.9% relative difference. The latter may be also observed, in a qualitative way, by the spatial extent of the electron/hole 1S orbitals, as shown in Figure S2c-1. The radial distribution function for the electron displays a slightly enhanced protrusion into the shell space, i.e. to the right of 1.35 nm mark, while that of the hole is virtually unaffected. Thus, shelling does not change the nature of the QD band character, which remains Type-I.

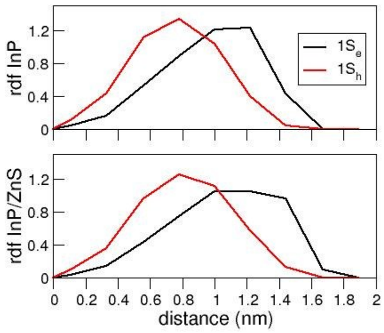

Figure S2e-1. Radial distribution functions of a 1S electron and hole for a 2.7 nm InP (upper) and InP with a 0.2 nm ZnS shell (lower) QDs.

We proceed with the calculation of all energy bound conduction and valence band states. These results are reported in Table S2e-2. Using these energies and the corresponding wavefunctions, we can recover a UV spectrum, which was also measured in the experiment. The energies of the transitions are calculated as $\Delta E = E_e - E_h - W_{eh}$, where W_{eh} is the electron-hole Coulomb interaction energy. The corresponding intensities which are calculated as oscillator strengths, are proportional to squared overlaps, S, multiplied by the transition energy E and the orbital degeneracy factor g, 1 for S, 3 for P, 5 for D, etc.

$$I \propto \Delta E g S^2 \qquad \text{S2e-4}$$

Note that only the transitions involving the same angular momentum values are allowed, i.e. $1S_h$->$1S_e$, $1P_h$->$1P_e$, $1P_h$->$2P_e$, $1D_h$->$1D_e$, etc. These spectra are depicted in Figure S2e-2.

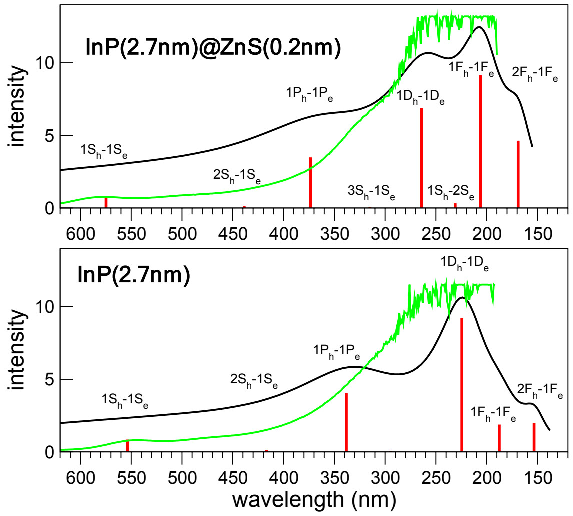

Figure S2e-2. UV absorption spectra of the 2.7 nm InP and its ZnS shelled version. The peaks, which are reported as oscillator strengths, are shown as vertical red bars, and their gaussian convolution is the solid black curve. The experiment is in green. The individual peaks are accompanied by their interband transition labels.

We first point out a good level of agreement between theory and experiment for the longer wavelength part of the spectra, ~300-600 nm, whereas the measurements are unreliable when $\lambda < 300$ nm. The calculated band origins appear close to where the experimental "bumps" are seen, near 550 nm for InP and 575 nm for InP@ZnS. The curves rise sharply at the shorter wavelengths, 350 nm, via the bright 1P-1P transitions. The calculated spectral intensities show further rise at 250 nm via the more intense 1D-1D transitions. The 1F-1F and 2F-1F are relatively weak in the InP dot, but are much brighter in the shelled dot. Analysis of the wavefunctions reveals a much stronger F-F overlaps in InP@ZnS. The most intense bands are 1D-1D in InP, ~220 nm, and 1F-1F in InP@ZnS, ~200 nm. A general conclusion that we can make upon inspection is that the spectra, measured and calculated, are quite similar between the pure and shelled InP dots, suggesting a relatively minor effect of the shell on the radiative interband transitions.

S2f. Estimation of the size of InP core and the ZnS shell thickness in the InP@ZnS$_{30mins}$ core/shell nanocrystals.

The average diameters of InP@ZnS$_{30mins}$ QDs used in the TA and PL studies was measured to be 2.9±0.2 nm by HAADF-TEM (Figure S11). Herein, we proceeded to estimate the size of the InP core in the core-shell structure by comparing the absorption spectra of InP aliquots taken right before injecting the sulfur precursor with the EMA calculation. Briefly, the EMA calculation shows that the InP QDs with a diameter of 2.7 nm can result in a bandedge exciton absorption at 550 nm, in agreement with the measured absorption spectrum of the InP core (Figure **S2e-2**). Meanwhile, Yu et. al previously measured that InP QDs with a

band edge exciton absorption at 525 nm corresponds to an averaged particle size of ~ 2.6 nm.[1] Thus, by combining the EMA calculation for the InP core and the TEM measurement of the core-shell structure, the shell thickness is estimated to be ~0.2 nm. Further EMA calculation of 2.7 nm InP core with a 0.2 nm ZnS shell thickness indeed replicate the absorption spectra of InP@ZnS (**S2e-2**), which supports the above estimation.

This estimation, however, does not reveal the distribution of the ZnS shell thickness in the final InP@ZnS QDs. As shown in Figure S11, the resulting InP@ZnS QDs have relatively narrow size distribution, with the standard deviation to be ~0.2 nm. Considering the thickness of one monolayer ZnS is around is ~0.3 nm,[13] we estimate that the shell thickness of ZnS in the InP@ZnS$_{30mins}$ should be approximate one monolayer.

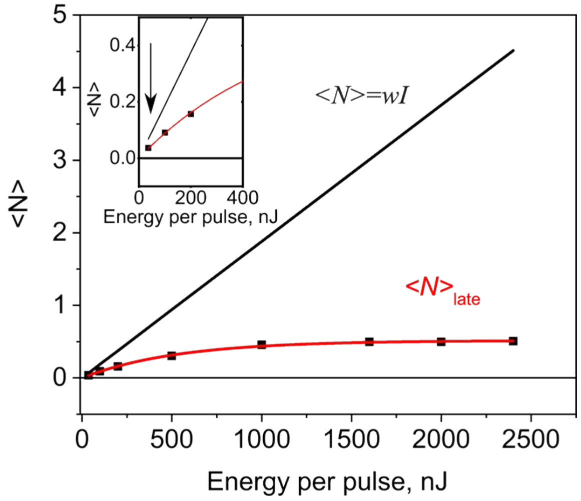

Figure S1. Calculated averaged exciton in nanocrystal as a function of excitation fluence for samples in Figure 1. The arrow marks the excitation power used in Figure 1, where the average number of excitons per quantum dot, <N>, is estimated to be ~ 0.07. **The calculation details are described into details on Page 7.**

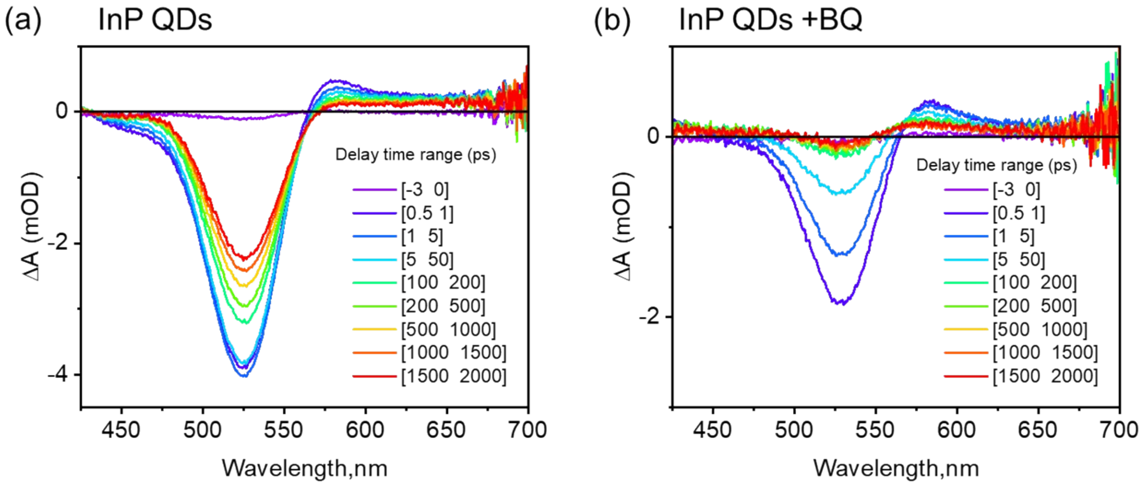

Figure S2. The TA spectra of InP (a) and after addition electron scavengers, benzoquinone (BQ) (b) Excitation Wavelength: 400 nm. Excitation Fluence: ~ 40 nJ/pulse. Time unit: ps.

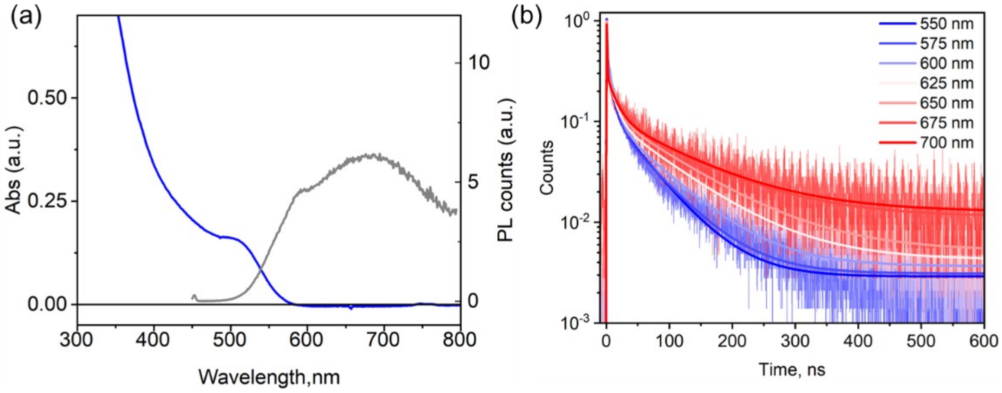

Figure S3. (a) UV-vis and PL spectra of InP QDs with the first exciton position at 525 nm (b) its PL decay when monitored at different wavelength after photoexcitation at 400 nm.

As shown in Figure S3b, the PL decay of InP (525 nm) differs when monitored at different wavelengths, where the red detection wavelengths demonstrate slower decay kinetics that that of the blue detection wavelengths. This result can be interpreted as the overlap of the band-edge exciton emission (blue region) and trap state assisted emission (red region), with similar kinetic behaviors reported previously in literature.[14]

In the Figure 2d, we used the PL decay at 550 nm to represent the PL decay of the band-edge exciton evidenced by (a) the PL emission spectrum (Figure S3a) appeared to be dominated by the band-edge exciton

emission. (b) the PL decay at 550 nm and 575 nm (blue lines, Figure S3b) show little difference, indicating the little impact of trap assisted emission at 550 nm.

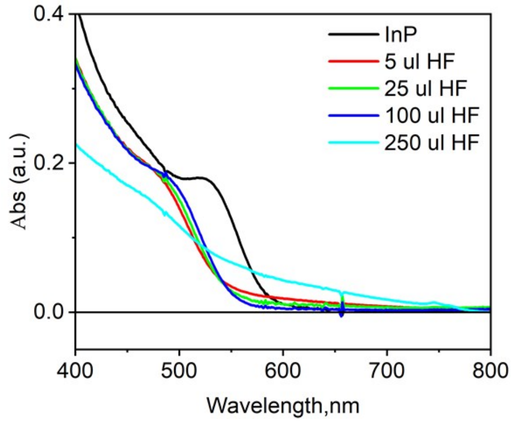

Figure S4. UV-Vis absorption spectra of InP QDs after treating with different amounts of HF.

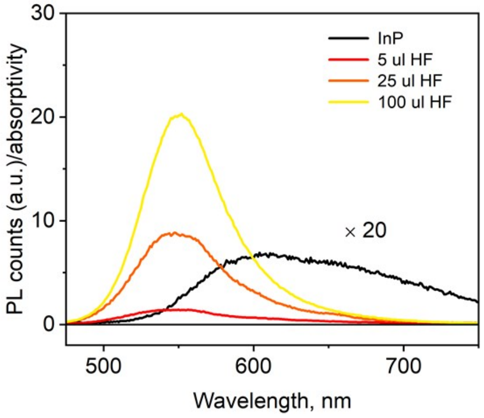

Figure S5. The normalized PL spectra of InP QDs after treating with different amounts of HF. Normalization is achieved by dividing the original PL counts by the absorptivity of the samples.

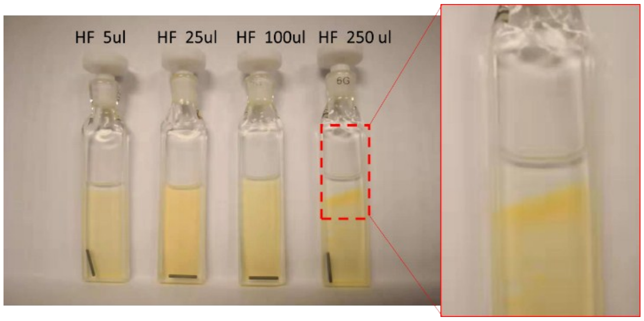

Figure S6. The visual appearance of InP QDs solution after treating with different amounts of HF.

When increasing the HF concentration above 250 µl, the QDs solution becomes aggregates and more scattering, also reflected in the UV-Vis measurement (Figure S1), and hinder the precise determination of its PLE.

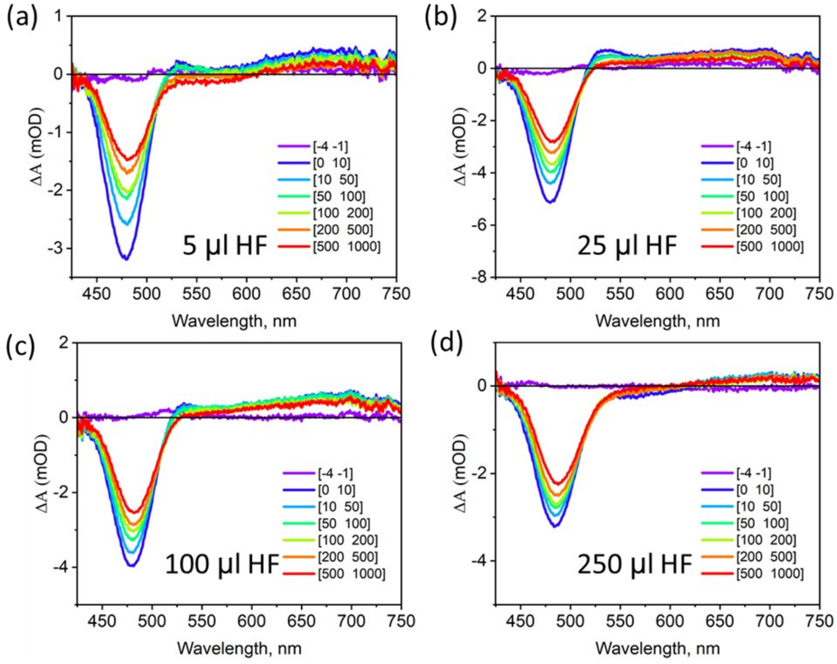

Figure S7. TA spectra of InP after treated with different amounts of HF. Excitation Wavelength: 400 nm. Excitation Fluence: ~ 160 nJ/pulse.

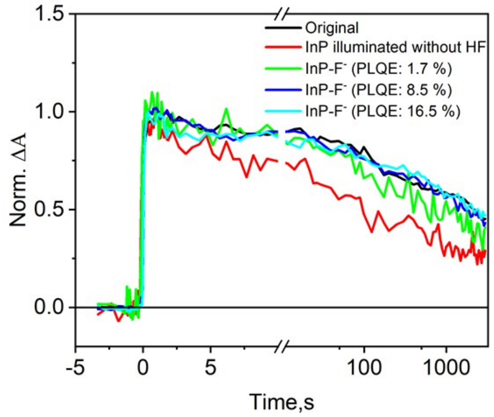

Figure S8. TAS measurement on a 2nd batch of InP QDs after treating with different amounts of HF. The red curve stands for the InP QDs sample treated under light condition with only the addition of TOPO but not HF. Excitation Wavelength: 400 nm. Excitation Fluence: ~40 nJ/pulse.

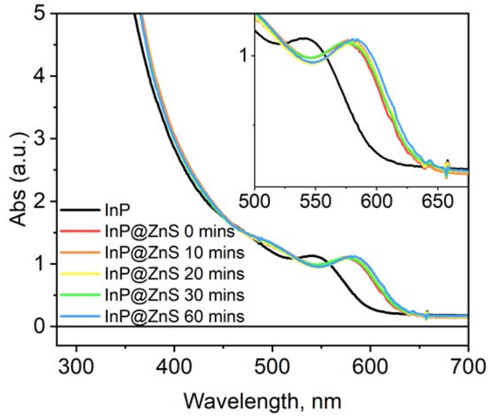

Figure S9. UV-Vis spectra of InP QDs as a function of the ZnS shell growth time. Inset: Zoomed-in absorption spectra region near the band edge.

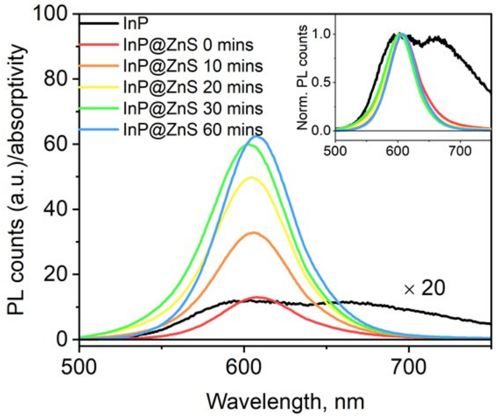

Figure S10. Normalized PL spectra of InP QDs as a function of the ZnS shell growth time. Normalization is done by dividing the original PL counts by the absorptivity of the samples. Inset: Normalized PL at the peak emission intensity.

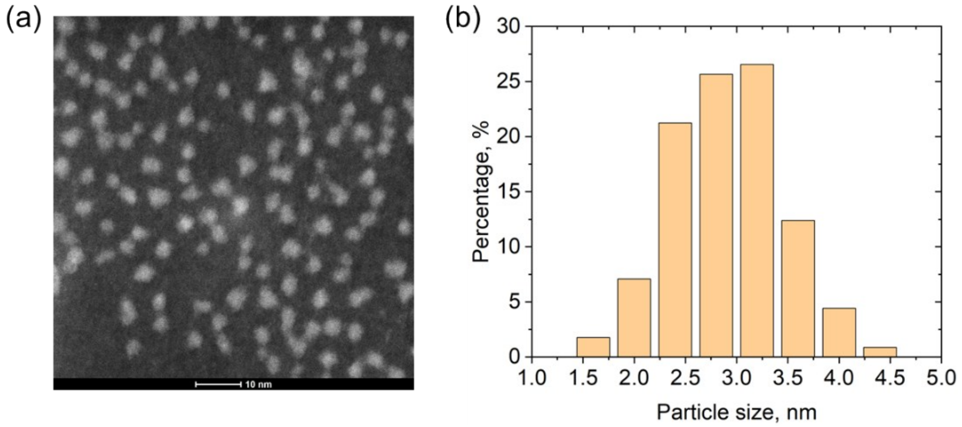

Figure S11. (a) HAADF-STEM picture of $InP@ZnS_{30mins}$ used in the present study. (b) the size distribution of nanoparticles extracted from Figure S11a.

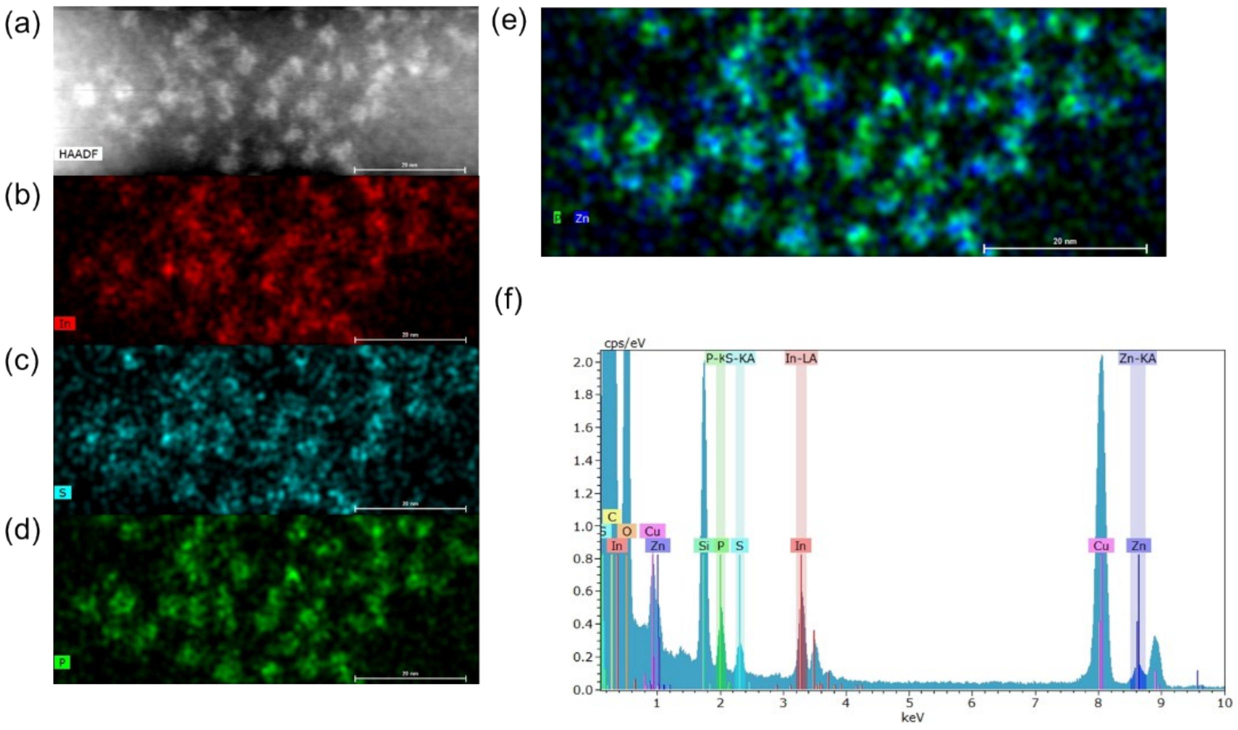

Figure S12. (a)-(f) the EDS mapping of $InP@ZnS_{30mins}$ used in the present study.

The STEM-EDS maps confirm the co-localization of zinc and sulfur with the InP particles and confirmed that there was no formation of ZnS shell-only particles in the sample. However, a core/shell structure could not be exclusively confirmed due to their small size and beam sensitivity. The previous study[15] showed that if only replacing the surface In^{3+} with Zn^{2+}, the resulting InP-Zn^{2+} QDs show almost identical absorption as that measured of the untreated InP QDs. The fact that we observed a significant shift (~40 nm) in the

absorption spectra of InP@ZnS indicates that the ZnS probably forms a shell structure around the InP QDs. This core-shell structure is further evidenced by Figure S2e-1, where the effective mass calculation (supplementary note S2e) shows that the core-shell structure can reproduce the absorption spectrum that agrees well with the measured results. Meanwhile, the ZnS shell is likely not uniform, considering the irregular shape of InP QDs and the very thin layer of ZnS shell.[16] This nonuniformity is believed to cause part of the InP QDs to have a similar biexciton decay lifetime as that of the untreated InP QDs (see Figure 5 in the main text and its discussion for details).

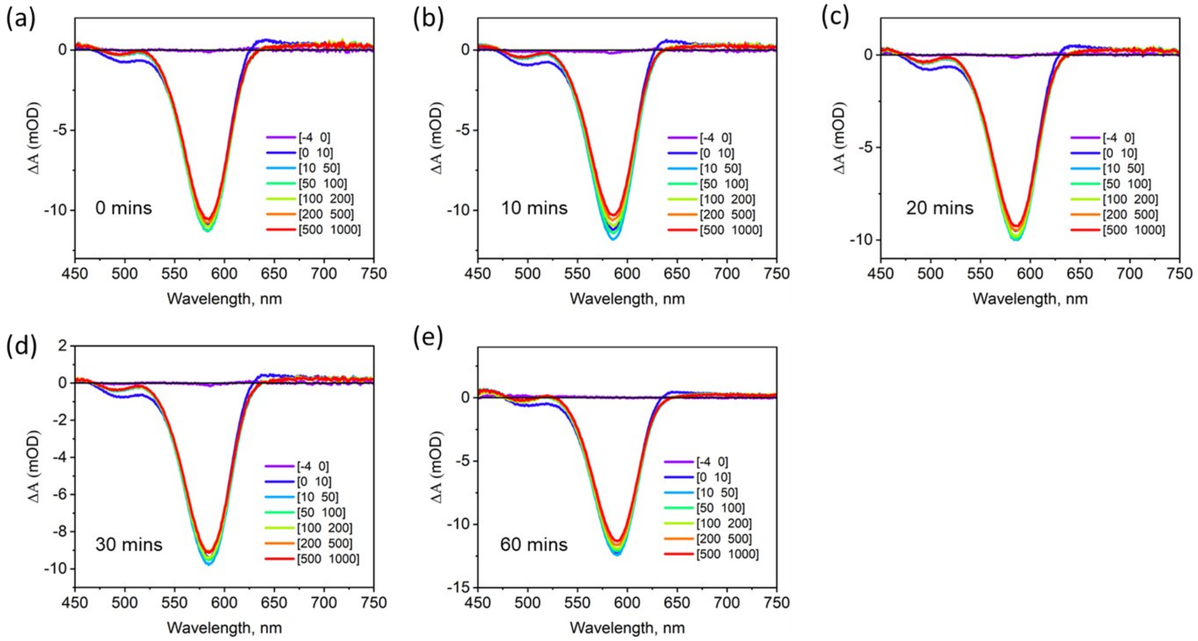

Figure S13. TA spectra of InP QDs with different ZnS shell growth times. Excitation Wavelength: 400 nm. Excitation Fluence: ~100 nJ/pulse. The spectra shown was averaged from the indicated time regions.

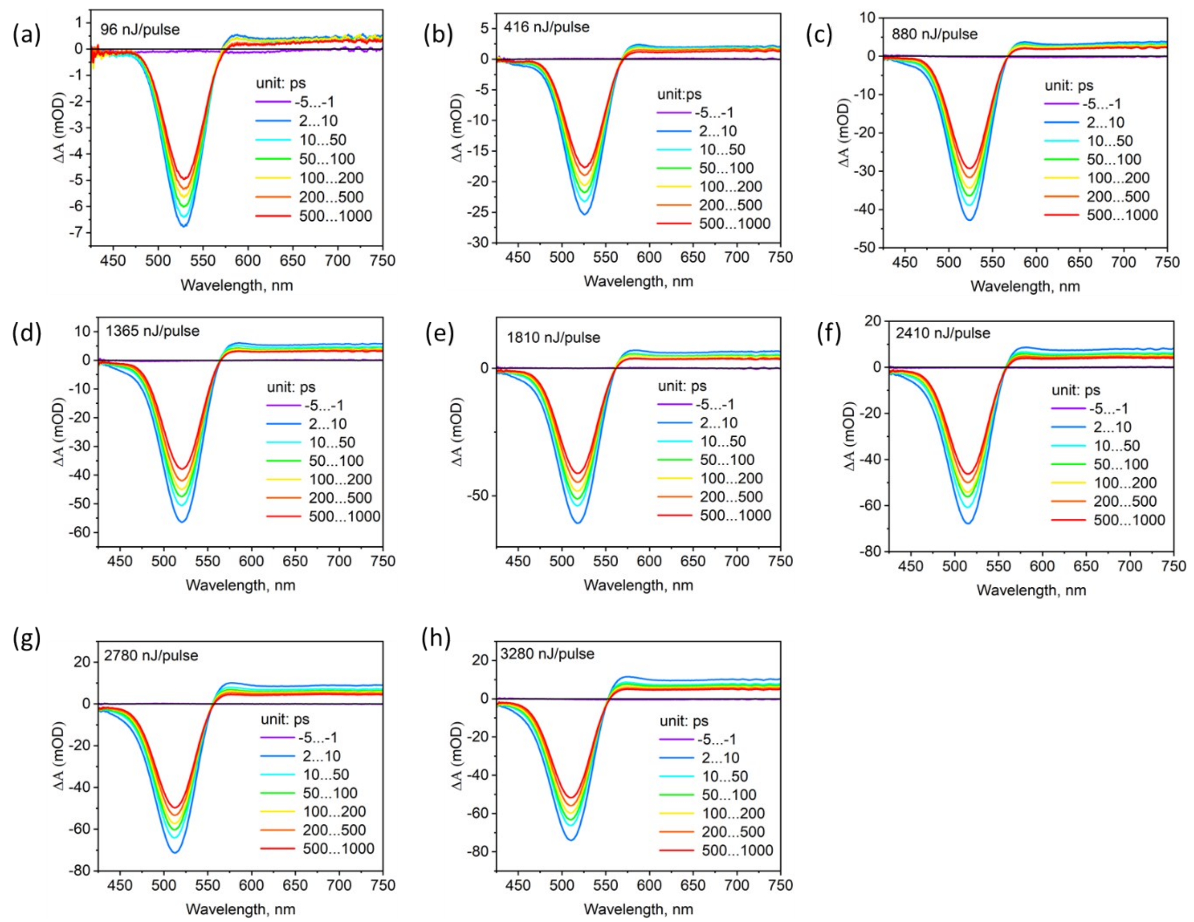

Figure S14. TA spectra of InP QDs after photoexcitation at 400 nm with different pump fluences. The spectra shown was averaged from the indicated time regions.1

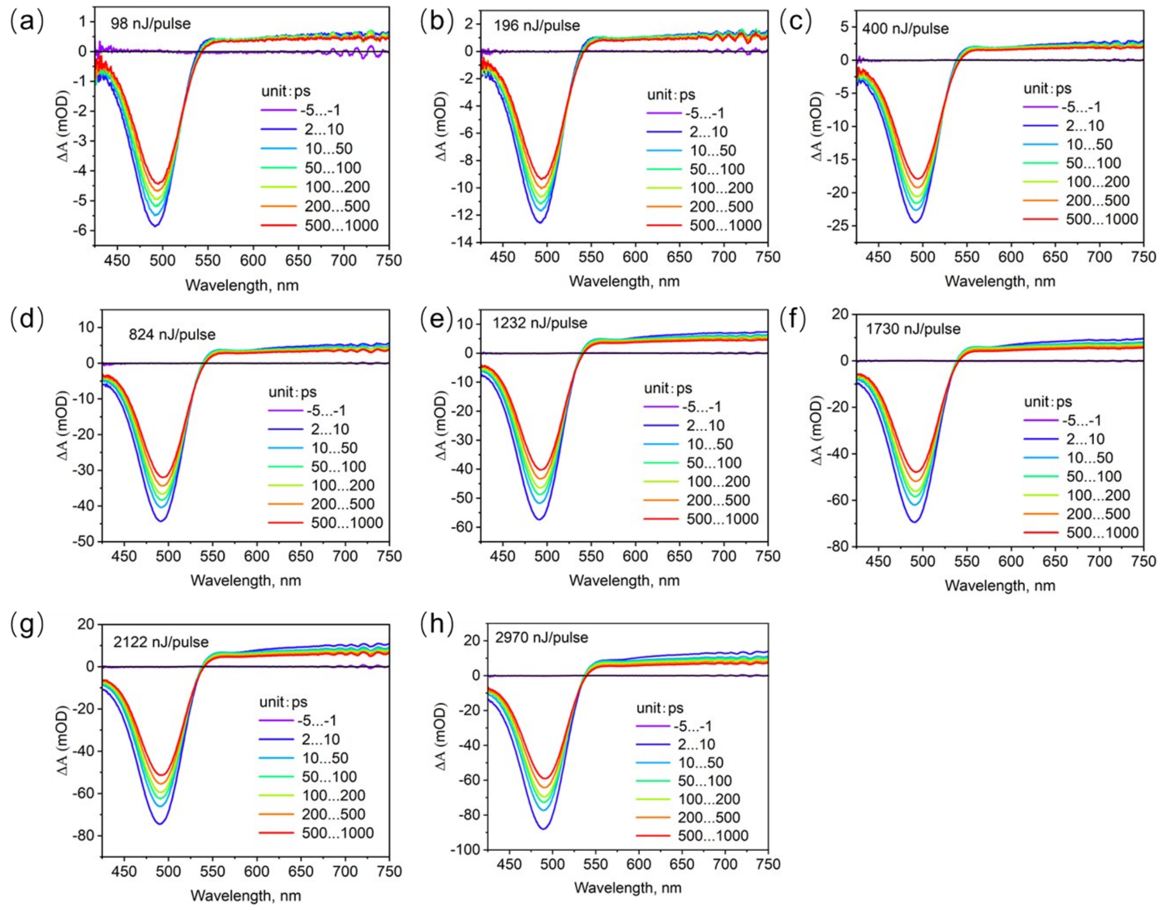

Figure S15. TA spectra of InP@$F^{-}_{100\mu l}$ QDs after photoexcitation at 400 nm with different pump fluences. The spectra shown was averaged from the indicated time regions.

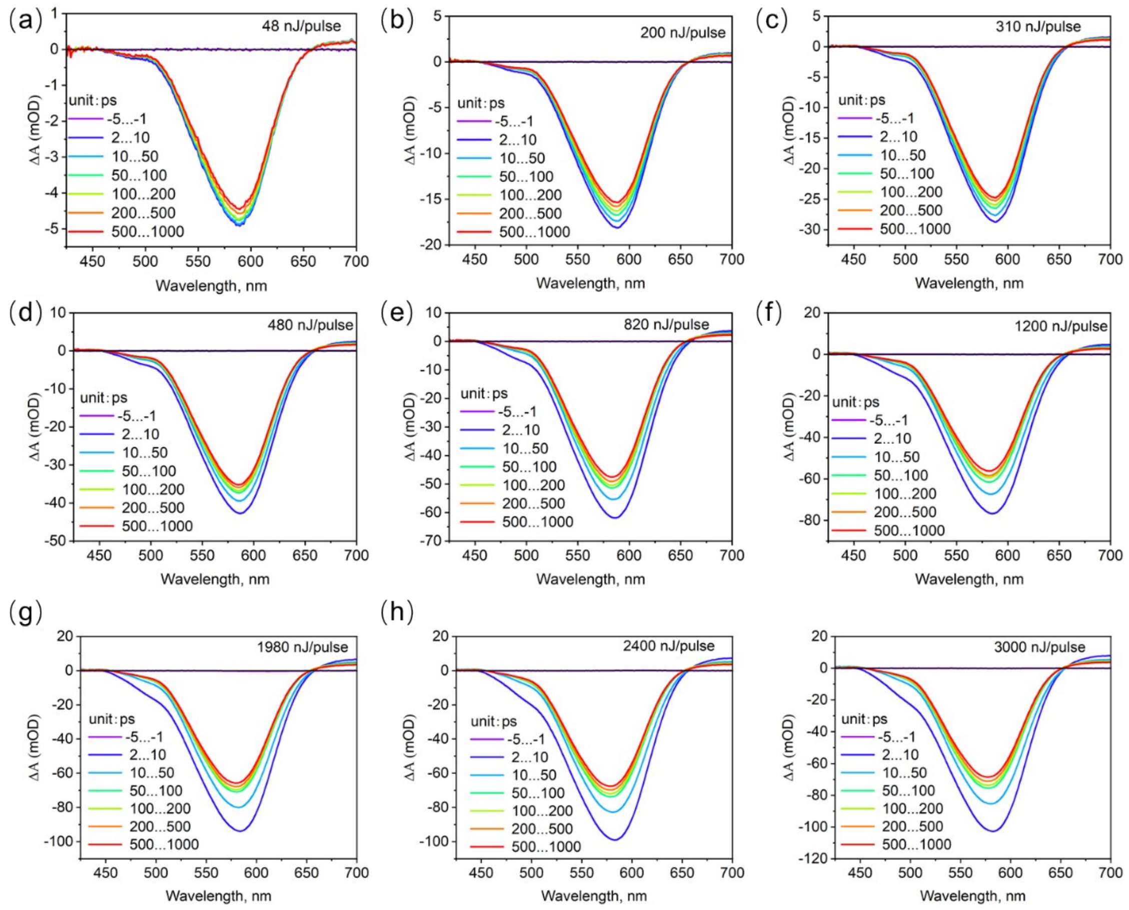

Figure S16. TA spectra of InP@ZnS after photoexcitation at 400 nm with different pump fluences. The spectra shown was averaged from the indicated time regions.

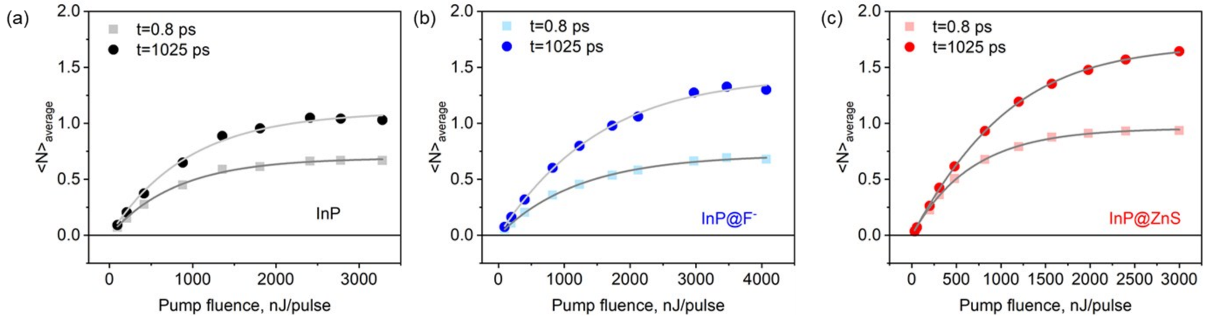

Figure S17. Calculated averaged exciton numbers as a function of excitation fluence for the InP, InP@$F^-_{100\mu l}$ and InP@ZnS30$_{mins}$ samples used in Figure S14–16, respectively. The calculation details are described in Supplementary Note S2c.

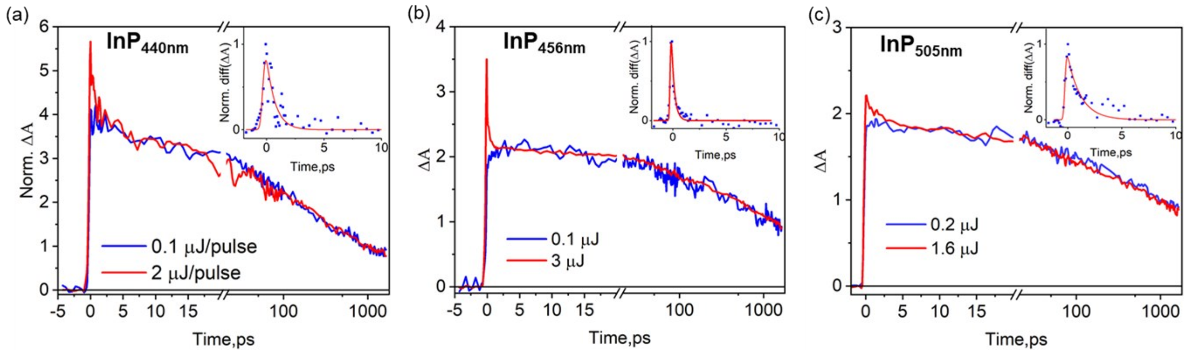

Figure S18. Biexciton Auger recombination processes of other InP QDs used in the present study. The normalized (at 1 ~ ns) XB decay kinetics of (a) InP_{440nm} (b) InP_{456nm} (c) InP_{505nm} QDs at low and high excitation fluence. The Inset show the corresponding normalized difference ΔA (Norm. diff(ΔA)), which is calculated by diff(ΔA)= $\Delta A_{high,fluence}$- $\Delta A_{low,fluence,}$ and their convoluted exponential fittings. Detailed description of the data processes can be found in Figure 5 of the main text. The original TA spectra and the fluence dependent XB decay kinetics are omitted.

Table S1 Fitting results of the XB and PL decays of the intrinsic InP QDs (Figure 2d)

	τ_1, ns (ratio)	τ_2, ns (ratio)	τ_3, ns (ratio)	τ_{weight}, ns
InP XB	3.5±0.6 (0.4±0.0)	19.4±4.8 (0.4±0.0)	84.3±14.7 (0.2±0.1)	26.8±3.5
InP PL	0.4±0.0 (0.8±0.0)	4.7±0.0 (0.2±0.0)	36.0±0.0 (0.1±0.0)	3.0±0.0

Table S2 Fitting results of the XB bleach and PL decay of the $InP@F^{-}_{100\mu l}$ sample (Figure 3d)

	τ_1, ns (ratio)	τ_2, ns (ratio)	τ_3, ns (ratio)	τ_{weight}, ns
$InP@F^{-}_{100\,\mu l}$ XB	18.2±4.9 (0.3±0.0)	3.6±0.7 (0.4±0.1)	94.1±14.2 (0.3±0.0)	32.4±4.5
$InP@F^{-}_{100\,\mu l}$ PL	2.1±0.0 (0.4±0.0)	14.5±0.2 (0.5±0.0)	60.3±0.0 (0.2±0.0)	18.1±0.1

Table S3 Fitting results of the XB bleach of InP@ZnS with different shell growth time (Figure 4b)

	τ_1, ns (ratio)	τ_2, ns (ratio)	τ_3, ns (ratio)	τ_{weight}, ns
InP@ZnS$_{0mins}$	4.1±0.4 (0.2±0.0)	29.0±2.0 (0.4±0.0)	174.1±10.0 (0.3±0.0)	70.0±3.1
InP@ZnS$_{10mins}$	5.9±0.5 (0.2±0.0)	41.7±3.7 (0.3±0.0)	260.6±17.8 (0.4±0.0)	110.8±7.2
InP@ZnS$_{20mins}$	4.6±0.5 (0.2±0.0)	36.9±2.5 (0.4±0.0)	299.7±18.7 (0.4±0.0)	128.6±7.5
InP@ZnS$_{30mins}$	6.2±0.6 (0.2±0.0)	42.8±3.4 (0.4±0.0)	296.4±20.1 (0.4±0.0)	124.5±8.1
InP@ZnS$_{60mins}$	6.3±0.5 (0.2±0.0)	47.5±3.2 (0.3±0.0)	367.5±24.7 (0.4±0.0)	156.7±9.9

Table S4 Fitting results of the PL decay of InP@ZnS with different shell growth time (Figure 4c)

	τ_1, ns (%)	τ_2, ns (%)	τ_3, ns (%)	τ_{weight}, ns
InP@ZnS0mins	4.1±0.1 (0.4±0.0)	39.3±0.4 (0.5±0.0)	182.8±1.4 (0.1±0.0)	45.5±0.3
InP@ZnS$_{10mins}$	6.1±0.1 (0.3±0.0)	41.4±0.4 (0.5±0.0)	182.8±1.4 (0.1±0.0)	49.4±0.2
InP@ZnS$_{20mins}$	8.1±0.2 (0.3±0.0)	42.5±0.4 (0.6±0.0)	182.8±1.4 (0.1±0.0)	50.6±0.3
InP@ZnS$_{30mins}$	8.9±0.2 (0.3±0.0)	43.7±0.4 (0.6±0.0)	182.8±1.4 (0.1±0.0)	46.3±0.2
InP@ZnS$_{60mins}$	5.3±0.0 (0.4±0.0)	39.3±0.0 (0.5±0.0)	182.8±0.0 (0.1±0.0)	37.3±0.0

Reference

1 S. Yu, X.-B. Fan, X. Wang, J. Li, Q. Zhang, A. Xia, S. Wei, L.-Z. Wu, Y. Zhou and G. R. Patzke, *Nat. Commun.*, 2018, **9**, 4009.

2 D. V. Talapin, N. Gaponik, H. Borchert, A. L. Rogach, M. Haase and H. Weller, *J. Phys. Chem. B*, 2002, **106**, 12659–12663.

3 M. D. Tessier, D. Dupont, K. De Nolf, J. De Roo and Z. Hens, *Chem. Mater.*, 2015, **27**, 4893–

4898.

4 J. Huang, Z. Huang, Y. Yang, H. Zhu and T. Lian, *J. Am. Chem. Soc.*, 2010, **132**, 4858–4864.

5 Q. Li and T. Lian, *Nano Lett.*, 2017, **17**, 3152–3158.

6 A. L. Kaledin, T. Lian, C. L. Hill and D. G. Musaev, *J. Chem. Theory Comput.*, 2014, **10**, 3409–3416.

7 A. L. Kaledin, T. Lian, C. L. Hill and D. G. Musaev, *J. Phys. Chem. B*, 2015, **119**, 7651–7658.

8 Y. Jia, J. Chen, K. Wu, A. Kaledin, D. G. Musaev, Z. Xie and T. Lian, *Chem. Sci.*, 2016, **7**, 4125–4133.

9 G. Allan, C. Delerue, M. Lannoo and E. Martin, *Phys. Rev. B*, 1995, **52**, 11982–11988.

10 J. D. Jackson, *Classical electrodynamics*, Wiley & Sons: New York, 1962.

11 X. Xiang, J. Fielden, W. Rodríguez-Córdoba, Z. Huang, N. Zhang, Z. Luo, D. G. Musaev, T. Lian and C. L. Hill, *J. Phys. Chem. C*, 2013, **117**, 918–926.

12 H. Zhu, N. Song and T. Lian, *J. Am. Chem. Soc.*, 2010, **132**, 15038–15045.

13 V. Gray, P. Xia, Z. Huang, E. Moses, A. Fast, D. A. Fishman, V. I. Vullev, M. Abrahamsson, K. Moth-Poulsen and M. Lee Tang, *Chem. Sci.*, 2017, **8**, 5488–5496.

14 Y. Kim, S. Ham, H. Jang, J. H. Min, H. Chung, J. Lee, D. Kim and E. Jang, *ACS Appl. Nano Mater.*, 2019, **2**, 1496–1504.

15 K. E. Hughes, J. L. Stein, M. R. Friedfeld, B. M. Cossairt and D. R. Gamelin, *ACS Nano*, 2019, **13**, 14198–14207.

16 N. Kirkwood, A. De Backer, T. Altantzis, N. Winckelmans, A. Longo, F. V. Antolinez, F. T. Rabouw, L. De Trizio, J. J. Geuchies, J. T. Mulder, N. Renaud, S. Bals, L. Manna and A. J. Houtepen, *Chem. Mater.*, 2020, **32**, 557–565.
